# Continuous Low-Thrust Maneuver Planning for Space Gravitational Wave Formation Reconfiguration Based on Improved Particle Swarm Optimization Algorithm

**DOI:** 10.3390/s23063154

**Published:** 2023-03-15

**Authors:** Zhenkun Lu, Jihe Wang, Xiaobin Lian, Juzheng Zhang, Yu Zhang, Jikun Yang

**Affiliations:** 1 MOE Key Laboratory of TianQin Mission, TianQin Research Center for Gravitational Physics & School of Physics and Astronomy, Frontiers Science Center for TianQin, Gravitational Wave Research Center of CNSA, Sun Yat-sen University (Zhuhai Campus), Zhuhai 519082, China; 2 School of Aeronautics and Astronautics, Sun Yat-sen University (Shenzhen Campus), Shenzhen 518107, China; 3 Shanghai Institute of Satellite Engineering, Shanghai 201109, China

**Keywords:** relative orbit elements, formation reconfiguration, maneuver planning, gravitational waves, particle swarm optimization

## Abstract

This study proposes a three-spacecraft formation reconfiguration strategy of minimum fuel for space gravitational wave detection missions in the high Earth orbit (10^5^ km). For solving the limitations of measurement and communication in long baseline formations, a control strategy of a virtual formation is applied. The virtual reference spacecraft provides a desired relative state between the satellites, which is then used to control the motion of the physical spacecraft to maintain the desired formation. A linear dynamics model based on relative orbit elements’ parameterization is used to describe the relative motion in the virtual formation, which facilitates the inclusion of J_2_, SRP, and lunisolar third-body gravity effects and provides a direct insight into the relative motion geometry. Considering the actual flight scenarios of gravitational wave formations, a formation reconfiguration strategy based on continuous low thrust is investigated to achieve the desired state at a given time while minimizing interference to the satellite platform. The reconfiguration problem is considered a constrained nonlinear programming problem, and an improved particle swarm algorithm is developed to solve this problem. Finally, the simulation results demonstrate the performance of the proposed method in improving the maneuver sequence distribution and optimizing maneuver consumption.

## 1. Introduction

Gravitational wave detection from space has been an active research area for decades. The Laser Interferometer Space Antenna (LISA) [1] was the first space-based gravitational wave detection project to be studied and evaluated. Since then, several innovative proposals have been put forward, including the ASTROD [2], DECIGO [3], OMEGA [4], TIANQIN [5], and TAIJI [6] projects. In these projects, three or more spacecraft are arranged in an equilateral triangle, and lasers are employed to measure the minute distance variations driven by gravitational waves. Compared with the ground detector, the space-based gravitational wave detector has the characteristics of a long baseline distance, fewer noise and interference sources, and the ability to detect gravitational waves from all directions, which make it especially suitable for capturing weaker gravitational wave signals in medium- and low-frequency bands [7]. However, space gravitational wave detectors present various challenges in their design and operation, among which the configuration maintenance and control of the precision formation flying is the most fundamental and urgent difficulty [1,8,9].

The primary obstacle is the extremely lengthy baseline distance, making communication and measurement difficult. For sufficient detection sensitivity, gravitational wave detectors in space need baseline distances of a few dozen kilometers or possibly millions of kilometers [10,11]. Nevertheless, such lengths would make it challenging to implement formation control methods that depend on relative state measurements. Some alternatives need to be considered, one option is to employ a mix of onboard sensors and external measurements to estimate the relative states of the spacecraft, and the other is to use model-based control methods [12]. Both strategies are effective at certain times, but the former will make the system more complex and expensive, and the latter will use up a lot of the onboard computational resources. Some academics have recently suggested a workable control method based on virtual formation. Lippe [13] offers a novel approach to minimizing delta-v for spacecraft swarm reconfiguration through refinement of the real or virtual chief spacecraft orbit, which is a closed orbit of arbitrary eccentricity. Sherrill [14] presents a simple method for roughly applying the HCW equations to the chiefs in an elliptic orbit; a virtual chief is described as a circularized version of the actual chief in the approach. Caruso [15] refines and extends a multiple impulse trajectory transfer method, which is based on the linearized CW equation and is solved in an optimal framework by minimizing the total velocity variation; in the study, a massless point covering a circular orbit around a certain celestial body was created as the formation’s (virtual) chief. Huang [16] proposed a spacecraft orbit correction method based on a virtual formation configuration design, and the correction was realized by four-pulse control. In view of the previous work, the virtual reference spacecraft provides a desired relative state between the satellites, which can significantly reduce the communication and measurement requirements between the spacecraft and make it easier to decouple the spacecraft’s motion in the formation, and in this study, we adopt this strategy.

An additional difficulty in a close-range decentralized control architecture based on virtual formations is formation reconfiguration under complicated constraints. Reconfiguration or modification is defined as transforming a spacecraft’s formation from an initial configuration to another desired configuration while also taking into account its capabilities, constraints, and objectives during the process [17]. It is commonly carried out utilizing optimization algorithms that evaluate different thrust scenarios and determine the most efficient and effective solution for a given mission. There is already a wealth of research on close formation reconfiguration control, ranging from continuous thrust to impulsive strategies, from analytical to numerical forms, and from using the relative Cartesian state to orbital element descriptions [18]. A typical class of research focuses on using impulsive thrust strategies. Vaddi [19] proposed an analytic, two-pulse control scheme for formation reconstruction and establishment. Michelle [20] presented a closed-form method for the fuel-optimal guidance and control of relative motion in the formation flying and rendezvous of spacecraft using impulsive maneuvers. Gaias [18] described an impulsive maneuvers planner for onboard autonomous optimal formation flying reconfigurations in a near-circular orbit. The impulse control strategy is an applicable and flexible control method; however, it is challenging to realize the relative velocity’s instantaneous change in practical engineering for a gravitational wave detection satellite equipped with μN-class thrusters. Continuous control is becoming popular in new space missions thanks to the advancement of microelectronic propulsion. Ben [21] derives a control concept for formation flight applications using analytical finite-duration approaches. Di Mauro [22,23,24] presents a solution to the minimum-fuel spacecraft formation reconfiguration maneuver in J_2_ perturbed near-circular orbits and subsequently investigates several flight scenarios by analytical and numerical methods. Zhang [25] proposes a control parameter direct optimization method for the optimal short-range elliptic orbit rendezvous problem using on–off constant thrust; during the study, the optimal control problem is transformed into a nonlinear programming problem with bound constraints on the optimization variables and terminal equality constraints. The aforementioned continuous control strategies can provide a reference for the reconfiguration control of a gravitational wave formation, but the current research mostly concentrates on near-ground scenarios; therefore, related research still needs to be refined and improved in order to be adaptable to the high Earth orbit environment while meeting the limitations of poor maneuverability, a long mission period, and high accuracy requirements in gravitational wave missions.

**Figure 1:**
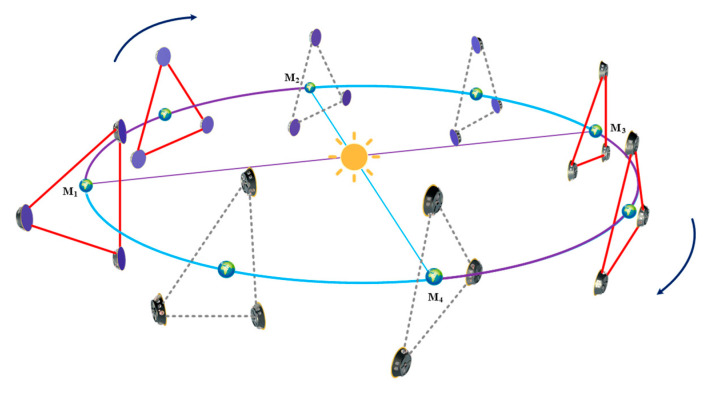
Operating modes of geocentric space gravitational wave detectors, where M1–M2, M3–M4 are scientific detection stages (represented by purple lines), and M2–M3 and M4–M1 are maintenance and adjustment stages (represented by cyan lines).

In this paper, a geocentric space gravitational wave detection mission is selected as a candidate for analysis. Compared with the heliocentric scheme, the geocentric scheme faces a more complex space environment and dynamics [9], thus posing a more pressing demand for the maintenance and control of the formation configuration. Figure 1 depicts the mission’s operational mode: Scientific gravitational wave detection will operate when the direction of the Sun is sufficiently angled with the orbital plane; when the direction of the Sun is almost parallel to the orbital plane, scientific observations will be put off for maintenance.

The main objective of this study is to develop a minimum-fuel spacecraft formation reconfiguration strategy for future space missions, such as space gravitational wave detectors, a distributed remote sensing cluster or solar satellite systems, etc. [5,8,26,27,28]. Firstly, a configuration reconfiguration scheme suitable for space gravitational wave detection formation is proposed, which is based on the concept of virtual formation. Virtual formation involves using a reference trajectory, or “virtual” formation, to guide the motion of the spacecraft in the formation; this approach can enable precise control of the formation configuration, even in the presence of uncertainty and perturbations. Secondly, the relative motion is described using a linear dynamics model based on the parameterization of relative orbit elements; compared with the commonly used Cartesian relative states, this description method can not only effectively reduce the error caused by linearization and facilitate the inclusion of perturbation effects, but it can also offer direct visualization of the effects of maneuvers on the relative orbit [22]. In addition, the model is built from a set of mean elements that slowly vary in time, which is beneficial for formation control since no additional fuel needs to be wasted to counteract short- and long-period effects caused by osculating elements [29]. Thirdly, a piecewise constant thrust formation reconfiguration mechanism is described. Simultaneous in-plane and out-plane controls are adopted to improve the reconfiguration efficiency; in order to avoid the long duration or frequent orbital maneuvers adding difficulty to the control of the test masses, the whole task interval is discretized. Finally, an improved particle swarm optimization algorithm is proposed to solve the multi-constrained maneuvers planning. The improved algorithm introduces measures such as adaptive factors, dynamic penalty functions, and updated rules for better global search performance. The innovation of this paper can be summarized as:

(1) A minimum-fuel reconfiguration strategy applicable to gravitational wave formations is proposed. The strategy can flexibly respond to different complex scenarios during gravitational wave missions with less interference to the satellite platform.
(2) Several measures are employed to improve the performance of the particle swarm algorithm. The improved particle swarm algorithm has better stability and superior global search capability when dealing with problems with complex constraints.

The outline of the paper is as follows. In Section 2, the concept of virtual formation, the relative motion model, and the reconfiguration control strategy are given; Section 3 introduces the maneuver planning process based on the improved particle swarm algorithm; the numerical simulation of the proposed control scheme is presented in Section 4; and finally, Section 5 draws the conclusion.

## 2. Formation Reconfiguration Control Strategy Based on Continuous Low Thrust

This part introduces the concept of virtual formation and the dynamic model describing the relative motion of the physical spacecraft relative to the virtual reference point.

### 2.1. Definition of the Coordinate System and Introduction of the Virtual Formation

**Figure 2:**
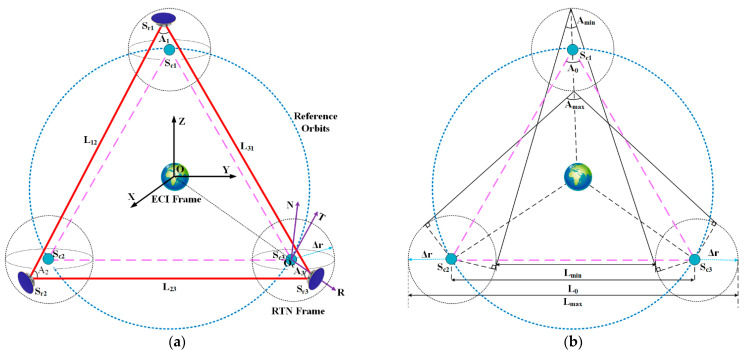
The illustration of the virtual formation. (**a**) Definition of the coordinate system; (**b**) geometric relationship between physical spacecraft in virtual formation. The bright red dotted lines and the red lines represent the nominal and real arm lengths between spacecraft, respectively. (Each satellite corresponds to an optimized nominal orbit. In order to express clearly, only one is drawn in the figure, as shown by the blue dotted line).

In order to facilitate the description of the spacecraft’s absolute motion and relative motion, the following coordinate system is defined in this paper, as shown in Figure 2.

(1) The Earth-centered inertial (ECI) reference frame: the origin *O* is located at the center of the earth, the OX axis points towards the vernal equinox, the OZ axis points towards the north celestial pole, and the OY axis is perpendicular to the XOZ plane and follows the right-hand rule.
(2) The Radial–Transversal–Normal (RTN) orbital frame: the $O_{c}R$ axis is aligned with the radial direction, pointing outward, the $O_{c}N$ axis is aligned with the orbit momentum vector, and the $O_{c}T$ axis completes the triad.

The illustration of the virtual formation is depicted in Figure 2a. The reference point operating on the nominal orbit is defined as the virtual chief spacecraft. The spacecraft traveling on the actual trajectory with errors is defined as the deputy spacecraft. As a result, the original long baseline formation is split into three close-proximity virtual formations, each comprising a physical spacecraft and its corresponding virtual reference point. As shown in the figure, $S_{c1}$, $S_{c2}$, and $S_{c3}$ represent virtual reference points, $S_{r1}$, $S_{r2}$, and $S_{r3}$ are physical spacecraft; $L_{ij}(i,j=1,2,3)$ is the arm length between spacecraft $S_{ri}$ and $S_{rj}$; $A_{i}(i=1,2,3)$ represents the breathing angle corresponding to spacecraft $S_{ri}$; and Δr is the envelope radius of the virtual formation configuration.

As discussed in reference [16], the maximum allowable envelope radius of the virtual formation can be calculated using direct planar geometry relationships when the difference in orbital elements between the physical spacecraft and the virtual reference point is small enough. The spatial geometric relationship between physical spacecraft is depicted in Figure 2b. In the figure, $L_{0}$, $L_{\min}$, and $L_{\max}$, respectively, represent the nominal arm length, the minimum arm length, and the maximum arm length; similarly, $A_{0}$, $A_{\min}$, and $A_{\max}$, respectively, represent the nominal breathing angle, the minimum breathing angle, and the maximum breathing angle.

The envelope radius of the virtual formation’s constraint conditions can be computed using the equilateral triangle configuration index and the geometric relationship depicted in Figure 2b:

(1) $$\{\begin{array}{l} 2\Delta r⩽\Delta L\\ A_{\max}-60^{{}^{\circ}}⩽\Delta A\\ 60^{{}^{\circ}}-A_{\min}⩽\Delta A \end{array}$$

In Equation (1), ΔL and ΔA are the arm length variation index and the breathing angle variation index, respectively, and the maximum breathing angle $A_{\max}$ and the minimum breathing angle $A_{\min}$ can be expressed as

$$\begin{array}{l} A_{\max}=2\arctan\left(\frac{\frac{\sqrt{3}}{2}L_{0}-\Delta r+2\Delta r\sqrt{1-\frac{\sqrt{3}\Delta r}{L_{0}}}}{\frac{3}{2}L_{0}-2\sqrt{3}\Delta r}\right)\\ A_{\min}=2\arctan\left(\frac{\frac{\sqrt{3}}{2}L_{0}+\Delta r-2\Delta r\sqrt{1+\frac{\sqrt{3}\Delta r}{L_{0}}}}{\frac{3}{2}L_{0}+2\sqrt{3}\Delta r}\right) \end{array}$$

With $\Delta L\ll L_{0}$, the terms containing $\frac{\Delta L}{L_{0}}$ can be ignored to simplify the formula further:

(2) $$\{\begin{array}{l} \Delta r⩽\frac{\Delta L}{2}\\ \Delta r⩽\frac{(3\tan\left(30^{{}^{\circ}}+\frac{\Delta A}{2}\right)-\sqrt{3})L_{0}}{2+4\sqrt{3}\tan\left(30^{{}^{\circ}}+\frac{\Delta A}{2}\right)}\\ \Delta r⩽\frac{(\sqrt{3}-3\tan\left(30^{{}^{\circ}}-\frac{\Delta A}{2}\right))L_{0}}{2+4\sqrt{3}\tan\left(30^{{}^{\circ}}-\frac{\Delta A}{2}\right)} \end{array}$$

By solving the system of inequality equations in Equation (2), the envelope boundary satisfying the gravitational wave detection requirement can be obtained.

### 2.2. Relative Motion Equation Modeling Based on Quasi-Nonsingular Relative Orbit Elements

In each virtual formation, the relative motion of the physical spacecraft relative to the virtual reference point can be described by a set of dimensionless relative orbital elements defined by D’Amico [30].

In the ECI reference frame, let $\alpha_{c}={\left[a_{c},e_{c},i_{c},\omega_{c},\Omega_{c},M_{c}\right]}^{T}$, $\alpha_{r}={\left[a_{r},e_{r},i_{r},\omega_{r},\Omega_{r},M_{r}\right]}^{T}$ denote the classical Keplerian orbital elements (OE) of the virtual reference point and the physical spacecraft, respectively. The quasi-nonsingular relative orbital elements (ROEs) are defined as

(3) $$\delta \alpha =\left(\begin{array}{c} \delta a\\ \delta \lambda \\ \delta e_{x}\\ \delta e_{y}\\ \delta i_{x}\\ \delta i_{y} \end{array}\right)=\left(\begin{array}{c} \delta a\\ \delta \lambda \\ \delta e\cos(\varphi )\\ \delta e\sin(\varphi )\\ \delta i\cos(\theta )\\ \delta i\sin(\theta ) \end{array}\right)=\left(\begin{array}{c} \left(a_{r}-a_{c}\right)/a_{c}\\ u_{r}-u_{c}+\left(\Omega_{r}-\Omega_{c}\right)\cos(i_{c})\\ e_{r}\cos(\omega_{r})-e_{c}\cos(\omega_{c})\\ e_{r}\sin(\omega_{r})-e_{c}\sin(\omega_{c})\\ i_{r}-i_{c}\\ \left(\Omega_{r}-\Omega_{c}\right)\sin(i_{c}) \end{array}\right)$$

where δa is the semi-major axis difference, which has been normalized by the chief semi-major axis, δλ is the relative mean argument of longitude, $\delta e={\left[\delta e_{x},\delta e_{y}\right]}^{T}$ is the relative eccentricity vector, and $\delta i={\left[\delta i_{x},\delta i_{y}\right]}^{T}$ is the relative inclination vector. Additionally, u=ω+M is the mean argument of latitude, the phase angles of φ and θ are termed the relative perigee and relative ascending node, respectively.

Assuming that the reference orbits in a near-circular reference orbit and the distance between the physical satellite and the virtual point is much smaller than the orbital radius of the virtual point, the ROEs can be written as functions of the integration constants of the HCW equations, The linear map [31,32] can be described as

(4) $$\delta x=Τ(u_{c})a_{c}\delta \alpha$$

where

$$Τ(u_{c})=\left[\begin{array}{cccccc} 1 & 0 & -\cos(u_{c}) & -\sin(u_{c}) & 0 & 0\\ 0 & 1 & 2\sin(u_{c}) & -2\cos(u_{c}) & 0 & 0\\ 0 & 0 & 0 & 0 & \sin(u_{c}) & -\cos(u_{c})\\ 0 & 0 & n_{c}\sin(u_{c}) & -n_{c}\cos(u_{c}) & 0 & 0\\ -\frac{3}{2}n_{c} & 0 & 2n_{c}\cos(u_{c}) & 2n_{c}\sin(u_{c}) & 0 & 0\\ 0 & 0 & 0 & 0 & n_{c}\cos(u_{c}) & n_{c}\sin(u_{c}) \end{array}\right]$$

**Figure 3:**
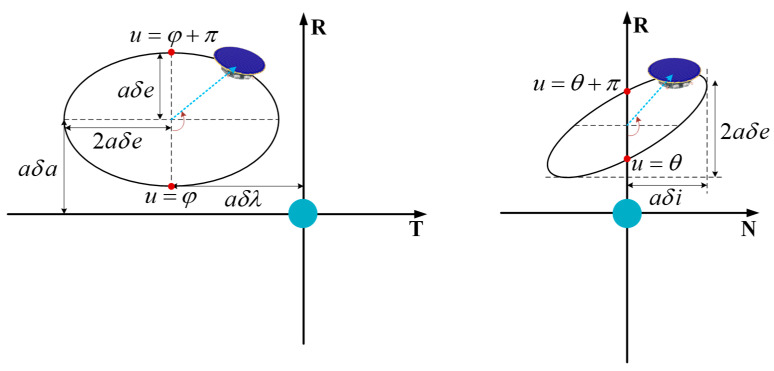
Geometric interpretation of quasi-nonsingular ROEs for near-circular orbits.

Accordingly, the inverse transformation can be easily realized by

(5) $$a_{c}\delta \alpha ={Τ}^{-1}(u_{c})\delta x$$

where $u_{c}$ is the latitude argument of the chief orbit at the instant $t_{c}$, which is interchangeable with time $t_{c}$ through the relation $u_{c}=u_{0}+W_{c}(t-t_{0})$, $W_{c}$ is the mean motion of the virtual reference point. $\delta x={\left[\delta r_{R},\delta r_{T},\delta r_{N},\delta r_{R},\delta v_{T},\delta v_{N}\right]}^{T}$ represents the relative position and velocity in the RTN reference frame. According to Equation (4), The ROEs will be decomposed into periodic motion in the RT plane and harmonic oscillations in the RN plane. In particular, the amplitudes of relative motion in *R*, *T*, and *N* directions are  aδe,  2aδe, and  aδi, respectively, offsets in *T* and *R* direction are given by  aδλ and  aδa, respectively. Moreover, the instantaneous phases of in-plane and out-of-plane motion are, respectively, represented by angle $u_{c}-\varphi$ and $u_{c}-\theta$, while relative phase angle θ−φ governs the orientation and shape of relative motion in the RN plane. The geometric insight provided by Equation (4) is illustrated in Figure 3.

### 2.3. Linearized Equations of Relative Motion with Perturbation

Consider a general absolute state $\alpha_{c}$ and relative state δα, a linear relative dynamic equation including perturbations and continuous thrust acceleration can be derived by using the relevant theory of Taylor expansion and Gaussian variational equation [20,33], which is given as

(6) $$\delta \dot{\alpha }(t)=A(\alpha_{c}(t))\cdot {\left[\delta \alpha (t),\;\Delta B_{srp}\right]}^{T}+[B(\alpha_{c}(t));\;0^{1\times 3}]\cdot u(t)$$

The term $A(\alpha_{c}(t))=A_{kep}+A_{J_{2}}(t)+A_{3rd}(t)+A_{srp}(t)$ is complete plant matrix, which is the superposition of Kepler motion and perturbation contributions such as the Earth’s oblateness effect J_2_, SRP, and lunisolar third body (T_3rdb_). The term B(t) represents the control input matrix and $u(t)={\left[u_{r},u_{t},u_{n}\right]}^{T}$ is the vector of constant control accelerations in the RTN orbital frame. $\Delta B_{srp}=B_{r,srp}-B_{c,srp}$ is the difference between the physical satellite and the chief SRP ballistic coefficient.

When the reference orbit is nearly circular, a simpler form can be derived by ignoring all terms that depend on eccentricity. These simplified plant matrices are given by

(7) $$A_{J_{2},nc}=k_{J_{2}}\left[\begin{array}{ccccccc} 0 & 0 & 0 & 0 & 0 & 0 & 0\\ -\frac{7}{2}E_{c}P_{c} & 0 & 0 & 0 & -F_{c}S_{c} & 0 & 0\\ 0 & 0 & 0 & -Q_{c} & 0 & 0 & 0\\ 0 & 0 & Q_{c} & 0 & 0 & 0 & 0\\ 0 & 0 & 0 & 0 & 0 & 0 & 0\\ \frac{7S_{c}}{2} & 0 & 0 & 0 & 2T_{c} & 0 & 0\\ 0 & 0 & 0 & 0 & 0 & 0 & 0 \end{array}\right]$$

The items of J_2_ plant matrix are as follows:

$$\begin{array}{l} \gamma_{{}_{J_{2}}}=\frac{3}{4}J_{2}R_{E}^{2}\sqrt{\mu },\;\kappa_{J_{2}}=\frac{\gamma_{J_{2}}}{a_{c}{}^{(7/2)}\eta^{4}},\;E_{c}=1+\eta ,\;F_{c}=4+3\eta ,\;G_{c}=\frac{1}{\eta^{2}},\;\\ Q_{c}=5\cos{\left(i_{c}\right)}^{2}-1,\;P_{c}=3\cos{\left(i_{c}\right)}^{2}-1,\;S_{c}=\sin(2i_{c}),\;T_{c}=\sin{\left(i_{c}\right)}^{2},\;\eta =\sqrt{1-e_{c}^{2}} \end{array}$$

where $R_{E}=6378.137\times 10^{3}\;(m)$, $\mu =3.986004415\times 10^{14}{\;(m}^{3}{/s}^{2})$, $J_{2}=1.08264\times 10^{-3}$.

(8) $$A_{srp,nc}=\kappa_{srp}\cdot \left[\begin{array}{ccccccc} 0 & 0 & 0 & 0 & 0 & 0 & 0\\ 0 & 0 & -2T_{s}{}_{{}_{1}} & 2T_{s_{2}} & 0 & 0 & 0\\ \frac{\eta }{2}T_{s_{2}} & 0 & 0 & 0 & -\eta T_{s_{3}} & \eta \cot(i_{c})T_{s_{1}} & \frac{\eta }{B_{srp}}T_{s_{2}}\\ \frac{\eta }{2}T_{s_{1}} & 0 & 0 & 0 & 0 & -\frac{\eta }{\sin{\left(i_{c}\right)}^{2}}T_{s_{4}} & \frac{\eta }{B_{srp}}T_{s_{1}}\\ 0 & 0 & \frac{1}{\eta }T_{s_{3}} & 0 & 0 & 0 & 0\\ 0 & 0 & 0 & \frac{1}{\eta }T_{s_{3}} & 0 & 0 & 0\\ 0 & 0 & 0 & 0 & 0 & 0 & 0 \end{array}\right]$$

The items of SRP plant matrix are as follows:

$$\begin{array}{l} \gamma_{srp}=\frac{3F_{srp}}{2\sqrt{\mu }},\;\kappa_{srp}=\gamma_{srp}B_{srp}\sqrt{a_{c}},\;y_{s}=\sin(\lambda_{s})\cos(i_{c}),\;z_{s}=\sin(\lambda_{s})\sin(i_{c})\\ X_{s_{1}}=\cos\left(\Omega_{c}-\lambda_{s}\right),\;Y_{s_{1}}=\sin\left(\Omega_{c}-\lambda_{s}\right)\cos(i_{c}),\;Z_{s_{1}}=\sin\left(\Omega_{c}-\lambda_{s}\right)\sin(i_{c})\\ X_{s_{2}}=\cos\left(\Omega_{c}+\lambda_{s}\right),\;Y_{s_{2}}=\sin\left(\Omega_{c}+\lambda_{s}\right)\cos(i_{c}),\;Z_{s_{2}}=\sin\left(\Omega_{c}+\lambda_{s}\right)\sin(i_{c})\\ Ts_{1}=\cos{\left(\frac{\varepsilon }{2}\right)}^{2}X_{s_{1}}+\sin{\left(\frac{\varepsilon }{2}\right)}^{2}X_{s_{2}},\;T_{s_{2}}=\cos{\left(\frac{\varepsilon }{2}\right)}^{2}Y_{s_{1}}+\sin{\left(\frac{\varepsilon }{2}\right)}^{2}Y_{s_{2}}-\sin(\varepsilon )z_{s}\\ T_{s_{3}}=\cos{\left(\frac{\varepsilon }{2}\right)}^{2}Z_{s_{1}}+\sin{\left(\frac{\varepsilon }{2}\right)}^{2}Z_{s_{2}}+\sin(\varepsilon )y_{s},\;T_{s_{4}}=\cos{\left(\frac{\varepsilon }{2}\right)}^{2}Z_{s_{1}}+\sin{\left(\frac{\varepsilon }{2}\right)}^{2}Z_{s_{2}} \end{array}$$

where $F_{srp}=-\frac{P_{sun}}{c}{\left(\frac{a_{sun}}{r_{sun}}\right)}^{2}$, with $P_{sun}=1367\;(W\cdot m^{-2})$ being the solar flux at 1 AU from the Sun, $c=3\times 10^{8}\;(m/s)$ the light speed, $\frac{a_{sun}}{r_{sun}}\approx 1$; $B_{srp}=\frac{C_{r}A_{srp}}{m}$ is the spacecraft ballistic coefficient, with $C_{r}$ being the reflectivity coefficient, $A_{srp}$ the cross-sectional area perpendicular to the Sun’s direction, and m the satellite mass; ε=23.44° is the ecliptic obliquity, $\lambda_{s}=\lambda_{0}+n_{s}t$ is the value of the ecliptic longitude of the Sun at the instant t, $n_{s}=2\pi /year$.

(9) $$\begin{array}{l} A_{3rdb,nc}=\kappa_{3rdb}. \end{array}\left[\begin{array}{ccccccc} 0 & 0 & 0 & 0 & 0 & 0 & 0\\ 0 & 0 & 0 & 0 & 2T_{d_{2}} & -2T_{d_{1}} & 0\\ 0 & 0 & 0 & \frac{2T_{d_{2}}}{\tan(i_{c})} & 0 & 0 & 0\\ 0 & 0 & -\frac{2T_{d_{2}}}{\tan(i_{c})} & 0 & 0 & 0 & 0\\ 3T_{d_{1}} & 0 & 0 & 0 & T_{d_{5}} & T_{d_{7}} & 0\\ 3T_{d_{2}} & 0 & 0 & 0 & 2(T_{d_{6}}-T_{d_{4}}) & 2T_{d_{8}}+T_{d_{3}} & 0\\ 0 & 0 & 0 & 0 & 0 & 0 & 0 \end{array}\right]$$

The items of T_3rdb_ plant matrix are as follows:

$$\begin{array}{l} \gamma_{3rdb}=\frac{3\mu_{3}}{4r_{3}^{3}\sqrt{\mu }},\;\kappa_{3rdb}=\frac{\gamma_{3rdb}\sqrt{a_{c}{}^{3}}}{\eta }\\ A_{3rdb}=\cos\left(\Omega_{c}-\Omega_{3}\right)\cos\left(u_{3}\right)+\cos\left(i_{3}\right)\sin\left(u_{3}\right)\sin\left(\Omega_{c}-\Omega_{3}\right)\\ B_{3rdb}=\cos(i_{c})\left[-\sin\left(\Omega_{c}-\Omega_{3}\right)\cos\left(u_{3}\right)+\cos\left(i_{3}\right)\sin\left(u_{3}\right)\cos\left(\Omega_{c}-\Omega_{3}\right)\right]+\sin(i_{c})\sin\left(i_{3}\right)\sin\left(u_{3}\right)\\ C_{3rdb}=\sin(i_{c})\left[\cos\left(u_{3}\right)\sin\left(\Omega_{c}-\Omega_{3}\right)-\cos\left(i_{3}\right)\sin\left(u_{3}\right)\cos\left(\Omega_{c}-\Omega_{3}\right)\right]+\cos(i_{c})\sin\left(i_{3}\right)\sin\left(u_{3}\right)\\ B_{\delta i_{x}}=\frac{\partial B_{3rdb}}{\partial \delta i_{x}}=-\sin(i_{c})\left[-\sin\left(\Omega_{c}-\Omega_{3}\right)\cos(u_{3})+\cos(i_{3})\sin(u_{3})\cos\left(\Omega_{c}-\Omega_{3}\right)\right]+\cos(i_{c})\sin(i_{3})\sin(u_{3})\\ C_{\delta i_{x}}=\frac{\partial C_{3rdb}}{\partial \delta i_{x}}=\cos(i_{c})\left[\cos(u_{3})\sin\left(\Omega_{c}-\Omega_{3}\right)-\cos(i_{3})\sin(u_{3})\cos\left(\Omega_{c}-\Omega_{3}\right)\right]-\sin(i_{c})\sin(i_{3})\sin(u_{3})\\ A_{\delta i_{y}}=\frac{\partial A_{3rdb}}{\partial \delta i_{y}}=-\frac{\sin\left(\Omega_{c}-\Omega_{3}\right)}{\sin(i_{c})}\cos(u_{3})+\cos(i_{3})\sin(u_{3})\frac{\cos\left(\Omega_{c}-\Omega_{3}\right)}{\sin(i_{c})}\\ B_{\delta i_{y}}=\frac{\partial B_{3rdb}}{\partial \delta i_{y}}=\cos(i_{c})\left[-\frac{\cos\left(\Omega_{c}-\Omega_{3}\right)}{\sin(i_{c})}\cos(u_{3})-\cos(i_{3})\sin(u_{3})\frac{\sin\left(\Omega_{c}-\Omega_{3}\right)}{\sin(i_{c})}\right]\\ C_{\delta i_{y}}=\frac{\partial C_{3rdb}}{\partial \delta i_{y}}=\sin(i_{c})\left[\cos(u_{3})\frac{\cos\left(\Omega_{c}-\Omega_{3}\right)}{\sin(i_{c})}+\cos(i_{3})\sin(u_{3})\frac{\sin\left(\Omega_{c}-\Omega_{3}\right)}{\sin(i_{c})}\right]\\ T_{d_{1}}=C_{3rdb}A_{3rdb},\;T_{d_{2}}=C_{3rdb}B_{3rdb},\;T_{d_{3}}=\frac{T_{d1}}{\tan(i_{c})},\;T_{d_{4}}=\frac{T_{d2}}{\tan(i_{c})},\;T_{d_{5}}=C_{\delta i_{x}}A_{3rdb}\\ T_{d_{6}}=C_{\delta i_{x}}B_{3rdb}+C_{3rdb}B_{\delta i_{x}},\;T_{d_{7}}=C_{\delta i_{y}}A_{3rdb}+C_{3rdb}A_{\delta i_{y}},\;T_{d_{8}}=C_{\delta i_{y}}B_{3rdb}+C_{3rdb}B_{\delta i_{y}} \end{array}$$

In the Sun third body perturbation, we have

$$\mu_{3}=1.327124\times 10^{20}\;(m^{3}/s^{2}),\;r_{s}=149597870\times 10^{3}\;(m),\;i_{3}=\varepsilon ,\;\Omega_{3}=0{}^{\circ},\;u_{3}=\lambda_{s}$$

In the Moon third body perturbation, parameters can be given as follows

$$\begin{array}{l} \mu_{3}=4.90280058\times 10^{12}\;(m^{3}/s^{2}),\;r_{3}=384403\times 10^{3}\;(m),\;\alpha =5.145{}^{\circ}\\ \Omega_{m}=\Omega_{0}-0.05295(t/86400),\;T=\left(JD-2451545\right)/36525,\;\\ \lambda_{m}=218.31617+481267.88088\times T-4.06\times T^{2}/3600\\ i_{3}=arccos(\cos(\varepsilon )\cos(\alpha )-\sin(\varepsilon )\sin(\alpha )\cos(\Omega_{m}))\;\\ \Omega_{3}=arc\sin(\frac{\sin(\alpha )\sin(\Omega_{m})}{\sin(i_{3})}),\;u_{3}=\lambda_{m}-\Omega_{m}+\arcsin(\frac{\sin(\varepsilon )\sin(\Omega_{m})}{\sin(i_{3})}) \end{array}$$

with $\Omega_{0}$ being the longitude of the mean ascending node of the lunar at the initial moment, JD is the Julian date at the instant $t_{c}$.

(10) $$B_{nc}=\frac{1}{a_{c}n_{c}}\left[\begin{array}{ccc} 0 & 2 & 0\\ -2 & 0 & 0\\ \sin(u_{c}) & 2\cos(u_{c}) & 0\\ -\cos(u_{c}) & 2\sin(u_{c}) & 0\\ 0 & 0 & \cos(u_{c})\\ 0 & 0 & \sin(u_{c}) \end{array}\right]$$

where the parameters $a_{c}$, $n_{c}$, and $u_{c}$ are defined as above.

Notably, each plant matrix in $A(\alpha_{c}(t))$ is independent and can be uniformly formalized into a [7 × 7] matrix, with the upper left [6 × 6] block containing the terms that relate the ROE with their variations (both conservative and nonconservative perturbing effects), and the upper right [6 × 1] column contains the linear relations that exist in-between the ROE variations and the ballistic coefficient difference (only nonconservative perturbing effects). Through examination of the rows of null matrices, it is possible to identify the ROE (regions of influence) that are not impacted by a particular perturbation. For detailed information on the plant matrix for the above items, we recommend that the reader read the literature’s appendixes [34]. Similarly, in the control matrix $B_{nc}$, in-plane and out-of-plane control are decoupled, which can be described as the radial (R) and tangential (T) direction maneuvers, represented by the first two columns, affecting only the in-plane ROE (${\left[\delta \alpha ,\delta \lambda ,\delta e_{x},\delta e_{y}\right]}^{T}$), and the normal (N) direction maneuvers, represented by the third column, which affect only the out-of-plane ROE (${\left[\delta i_{x},\delta i_{y}\right]}^{T}$). Meanwhile, the model has neglected factors such as eclipses effects, planetary gravity, and higher-order geopotential terms.

### 2.4. Converting from Osculating to Mean Elements

As mentioned above, the linear dynamic equations are based on the average state space, while the actual measurement information of the satellite is osculating states; therefore, the algorithm converting the osculating elements to the mean elements is necessary. Due to closed-form conversions between osculating to mean states under the variety of perturbations considered not being available in the literature, a numerical method of moving average filtering was applied.

**Figure 4:**
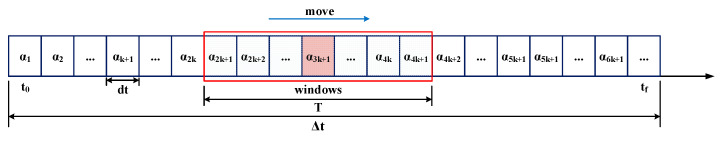
Schematic diagram of moving average filter algorithm. $t_{0}$, $t_{f}$ are the task beginning and end times, respectively, and dt is the interval time between each data.

The basic idea of the moving average filter algorithm is to set a fixed-width sliding window, which slides along the time series while taking the arithmetic mean of the data in the window as the output value. As shown in Figure 4, assuming that each orbital period has 2k+1 data, the average element corresponding to the $n_{th}$ osculating element is calculated by the following formula:

(11) $$\alpha_{m}(n)=\frac{1}{2k+1}\cdot \overset{i=k}{\underset{i=-k}{\sum }}\alpha (n+i)$$

This is a convenient and accurate method for the scenarios studied in this paper where the reference orbit is known.

## 3. Continuous Low-Thrust Maneuver Planning via Improved Particle Swarm Optimization

In this section, a general solution for formation reconfiguration using piecewise constant thrust is introduced. The problem is described as a nonlinear programming problem (NLP) with complex constraints, and then the maneuvering parameters are optimized and solved by an improved particle swarm optimization algorithm.

### 3.1. Reconfiguration Control Based on Piecewise Constant Thrust

In references [22,25], a tangential maneuvering strategy is used to obtain the minimum fuel consumption, which is an optimal strategy for the case where the eccentricity dominates the conformational divergence. In contrast to the studies described, δλ is the main divergence element in the study, which is caused by the geometrical differences of the orbits; for this reason, in-plane control simultaneously in the radial and tangential directions is a more efficient option. In addition, since the spacecraft contains two test masses, executing long-duration or short-term high-frequency maneuvers will make it more difficult to control the test masses and may even cause a collision with the satellite platform. Hence, a reasonable distribution of the thrust sequence is necessary.

**Figure 5:**
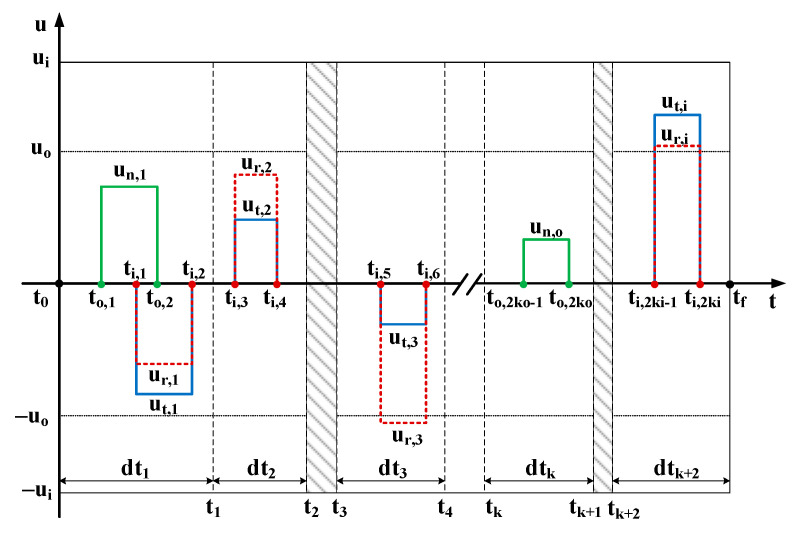
Piecewise constant acceleration profile of RTN reference frame. The red dotted line, the solid blue line, and the solid green line represent the thrust acceleration along the R-, T-, and N-axes, respectively, and the part filled with a gray slash indicates that orbital maneuver is prohibited during this period of time. The gray dashed line shows the dispersion to the task time.

As illustrated in Figure 5, it is assumed that after $k_{i}$ in-plane maneuvers and $k_{o}$ out-of-plane maneuvers, the formation reaches the desired state at the given time. The maximum amplitudes of the in-plane and out-of-plane thrusters are $u_{i}$ and $u_{o}$, respectively; each maneuver’s magnitude is expressed as $u_{j,k}\;(j=r,t,n\;and\;k=1,2,\ldots ,k_{i}\;or\;k_{o})$ and the maneuver duration is $\Delta t_{j}=t_{j,2k}-t_{j,2k-1}\;(k=1,2,\ldots ,k_{i}\;or\;k_{o})$. The whole task interval is discretized into $k_{i}$ and $k_{o}$ parts in terms of in-plane and out-of-plane according to the number of maneuvers and constraint limits, denoted as $\left[t_{k-1},t_{k}]\;(k=1,2,\ldots ,k_{i}\;or\;k_{o}\right)$. Then the process of converting the relative state $\delta \alpha (t_{0})$ at the time $t_{0}$ to the state $\delta \alpha (t_{f})$ at the time $t_{f}$ using the linear dynamic equation described in Section 2.3 can be specifically described as:

In the period $\left[t_{0},t_{o,1}\right]$, there is no maneuver acceleration, then the relative state $\delta \alpha (t_{o,1})$ at $t_{o,1}$ can be obtained by

(12) $$\delta \dot{\alpha }(t)=A(\alpha_{c}(t_{0}))\cdot {\left[\delta \alpha (t_{0}),\;\Delta B_{srp}\right]}^{T}$$

In the period $\left[t_{o,1},t_{i,1}\right]$, the constant thrust acceleration is ${\left[0,0,u_{n,1}\right]}^{T}$, then the relative state $\delta \alpha (t_{i,1})$ at $t_{i,1}$ can be obtained by

(13) $$\delta \dot{\alpha }(t)=A(\alpha_{c}(t_{o,1}))\cdot {\left[\delta \alpha (t_{o,1}),\;\Delta B_{srp}\right]}^{T}+[B(\alpha_{c}(t));\;0^{1\times 3}]\cdot {\left[0,0,u_{n,1}\right]}^{T}$$

Next, in-plane thrusters start working, the constant maneuver acceleration becomes ${\left[u_{r,1},u_{t,1},u_{n,1}\right]}^{T}$ in the period $\left[t_{i,1},t_{o,2}\right]$, then the relative state $\delta \alpha (t_{o,2})$ at $t_{o,2}$ can be obtained by

(14) $$\delta \dot{\alpha }(t)=A(\alpha_{c}(t_{i,1}))\cdot {\left[\delta \alpha (t_{i,1}),\;\Delta B_{srp}\right]}^{T}+[B(\alpha_{c}(t));\;0^{1\times 3}]\cdot {\left[u_{r,1},u_{t,1},u_{n,1}\right]}^{T}$$

Following this, in the interval $\left[t_{o,2},t_{i,2}\right]$, the constant thrust acceleration is ${\left[u_{r,1},u_{t,1},0\right]}^{T}$, then the relative state $\delta \alpha (t_{i,2})$ at $t_{i,2}$ can be obtained by

(15) $$\delta \dot{\alpha }(t)=A(\alpha_{c}(t_{o,2}))\cdot {\left[\delta \alpha (t_{o,2}),\;\Delta B_{srp}\right]}^{T}+[B(\alpha_{c}(t));\;0^{1\times 3}]\cdot {\left[u_{r,1},u_{t,1},0\right]}^{T}$$

Then again, all thrusters stop working in the interval $\left[t_{i,2},t_{i,3}\right]$, and the relative state $\delta \alpha (t_{i,3})$ at $t_{i,3}$ can be obtained by

(16) $$\delta \dot{\alpha }(t)=A(\alpha_{c}(t_{i,2}))\cdot {\left[\delta \alpha (t_{i,2}),\;\Delta B_{srp}\right]}^{T}$$

Similarly, by following the above rules in turn, the relative state $\delta \alpha (t_{f})$ at the end of the mission $t_{f}$ will be obtained, and the relative state satisfies

(17) $$\delta \alpha (t_{f})=\delta \alpha_{des}$$

The term $\delta \alpha_{des}$ is the desired mean ROE at the end of the maneuvering interval.

It has been demonstrated that fuel consumption saving is particularly important in practical space operations. In general, fuel consumption can be minimized by optimizing maneuvering parameters, which are handled in the following form:

Find: $u(t_{c})\subset {\mathbb{R}}^{3},\;t_{c}\in [t_{0},t_{f}]$

Minimizing: $J=\sum_{k_{i}=1}^{i}(\left|u_{r,k_{i}}\right|+\left|u_{t,k_{i}}\right|)\cdot (t_{i,2k_{i}}-t_{i,2k_{i}-1})+\sum_{k_{o}=1}^{o}\left|u_{n,k_{o}}\right|(t_{o,2k_{o}}-t_{o,2k_{o}-1})$

Dynamics constraints: $\delta \dot{\alpha }(t)=A(\alpha_{c}(t))\cdot {\left[\delta \alpha (t),\;\Delta B_{srp}\right]}^{T}+[B(\alpha_{c}(t));\;0^{1\times 3}]\cdot u(t)$

Boundary constraints: $\delta \alpha (t_{f})=\delta \alpha_{des}$

Maneuver magnitude constraint: $-u_{j}\leq u_{j}(t)\leq u_{j}\;(j=i,o)$

Maneuver time constraint: $t_{0}\leq t_{1}\leq t_{2}\leq \ldots \leq t_{2k-1}\leq t_{2}{}_{k}\leq t_{f}\;(k=1,2\ldots k_{i}\;\mathrm{or}\;ko)$

Other constraints: all kinds of sudden or planned maintenance work.

The problem above is known as a constrained nonlinear programming problem, where the optimization variables are the maneuver parameters $u(t_{c})$ and $t_{c}$, J is the objective function, and the remaining terms are the constraints.

### 3.2. Improved Particle Swarm Optimization Algorithm

Particle swarm optimization (PSO) is a population intelligence-based optimization algorithm. It simulates the search process of multiple candidate solutions in the form of particles and obtains the global optimal solution by continuously updating the velocity and position of the particles. However, when dealing with complex constraints or a large number of optimization variables, the algorithm often shows weaknesses of poor convergence and tends to fall into partial optimality. Considering the complexity of the problem in this paper, an improved particle swarm optimization algorithm is proposed.

The first part is to process the constraints. When applying particle swarm algorithms to solve optimization problems with constraints, the most generally used method is to add penalty terms to the objective function using the Lagrange multiplier method. The selection of penalty factors has been a difficult problem. If the penalty factor is too large, the algorithm tends to converge to a partial optimal; when the penalty factor is smaller, the algorithm may converge to an infeasible solution. In response to this question, a segmented penalty function that can be dynamically updated is introduced. The generalized objective function constructed by adding the penalty term is


(18) $$F(x)=f(x)+h(i_{c})p(x)$$

In the formula, $f(x)=J=\sum_{k_{i}=1}^{i}(\left|u_{r,k_{i}}\right|+\left|u_{t,k_{i}}\right|)\cdot (t_{i,2k_{i}}-t_{i,2k_{i}-1})+\sum_{k_{o}=1}^{o}\left|u_{n,k_{o}}\right|(t_{o,2k_{o}}-t_{o,2k_{o}-1})$ represents the sum of the in-plane and out-of-plane maneuver costs, $h(i_{c})=i_{c}\sqrt{i_{c}}$ is the penalty factor that updates dynamically with the number of iterations, $i_{c}$ is the current generation, and P(x) is the penalty term of the constraint, with

$$P(x)=\sum_{n=1}^{m}V(S_{n}(x))S_{n}(x)$$

$$S_{n}(x)=\max(0,S_{n}(x))$$

$$V(S_{n}(x))=\{\begin{array}{cc} c_{1} & S_{n}(x)<q_{1}\\ c_{2} & q_{1}\leq S_{n}(x)<q_{2}\\ \ldots & \ldots \\ c_{i-1} & q_{i-2}\leq S_{n}(x)<q_{i}{}_{-1}\\ c_{i} & S_{n}(x)\geq q_{i}{}_{-1} \end{array}$$

where $S_{n}(x)$ is the relative constraint penalty function, $V(S_{n}(x))$ is a segment function, $\left[c_{1},c_{2},\ldots c_{i}\right]$ and $\left[q_{1},q_{2},\ldots q_{i-1}\right]$ are penalty values and tolerances, respectively, whose amounts are set based on the specific problem.

Furthermore, in order to balance the conflict between minimizing the value of the generalized objective function and satisfying the constraints, by referring to Deb’s work [35], rules for evaluating the particle superiority are developed as follows:

(1) If both particles are feasible solutions, the particle with the smaller objective function is superior;
(2) If both particles are infeasible solutions, the particle with the more minor violation of the constraint is superior;
(3) When one of the two particles is a feasible solution, the particle that is feasible is chosen as the superior.

The other part is an improved update strategy for particles. The standard particle swarm optimization algorithm adopts constant inertia coefficients and learning factors; such a setup will lead to slow convergence of the algorithm at the end of evolution, and it will easily fall into partial optimality. Considering the above limitations, an adaptive variation mechanism is employed in this paper to improve the performance of the algorithm. The particle update formulas of the adaptive particle swarm optimization algorithm can be expressed as

(19) $$x(i_{c}+1)=x(i_{c})+v(i_{c}+1)$$

(20) $$v(i_{c}+1)=Wv(t)+C_{1}r_{1}\left(p_{\mathrm{best}\;}-x(i_{c})\right)+C_{2}r_{2}\left(g_{\mathrm{best}\;}-x(i_{c})\right)$$

where $i_{c}$ denotes the current generation; x and v are the position and velocity of the particle, respectively; W is the inertia coefficient; $C_{1}$ and $C_{2}$ are the learning factors; $r_{1}$ and $r_{2}$ are the random numbers distributed between [0,1]; $p_{\mathrm{best}\;}$ is the optimal solution of the particle itself; and $g_{\mathrm{best}\;}$ is the global optimal solution. W, $C_{1}$, and $C_{2}$ will dynamically adjust according to the following mechanism:

(21) $$W=\left(\omega_{\max}-\omega_{\min}\right)\times \exp{\left(-\varsigma \times \left(\frac{i_{c}}{I_{\max}}\right)\right)}^{2}$$

(22) $$C_{1}=c_{1}{,}_{\max}-\frac{\left(c_{1}{,}_{\max}-c_{1}{,}_{\min}\right)\times i_{c}}{I_{\max}}$$

(23) $$C_{2}=c_{2}{,}_{\min}+\frac{\left(c_{2}{,}_{\max}-c_{2}{,}_{\min}\right)\times i_{c}}{I_{\max}}$$

where $\omega_{\max}$ and $\omega_{\min}$ denote the maximum and minimum inertia coefficients, respectively; $c_{1}{,}_{\max},c_{2}{,}_{\max}$ and $c_{1}{,}_{\min},c_{2}{,}_{\min}$ are the maximum and minimum learning factors, respectively; $I_{\max}$ is the maximum iteration number; and ς(ς∈[25,55]) is an empirical parameter.

Using the update strategy described in Equations (21)–(23), the inertia coefficient W and the self-learning factor $C_{1}$ will decrease with increasing iterations, and the group learning factor $C_{2}$ will increase with increasing iterations. This adjustment mechanism will give the algorithm a strong global search capability in the early stage and a robust local search capability in the later phase.

Additionally, we introduce a random mutation factor after the update of particles in each generation to avoid the algorithm from falling into premature convergence, whose basic idea is to generate a random number $r_{d}(r_{d}\in [0,1])$ for each generation and compare it with a set mutation probability $\phi_{c}(\phi_{c}\in [0,1])$, which is set to 0.85 in this study. If the random number is larger than the probability, then:

(24) $$i=\mathrm{int}(\dim\times r_{d})$$

(25) $$x(i,j)=x_{\min}(i)+(x_{\max}(i)-x_{\min}(i))r_{d}$$

where, dim is the dimension of the optimization variable, i is the *i*th variable, j is the *j*th particle, and $x_{\min},x_{\max}$ are the boundary values of the *i*th variable.

In this way, each iteration has a certain possibility to randomly change the value of a certain individual, which will improve the whole particle swarm’s performance.

**Figure 6:**
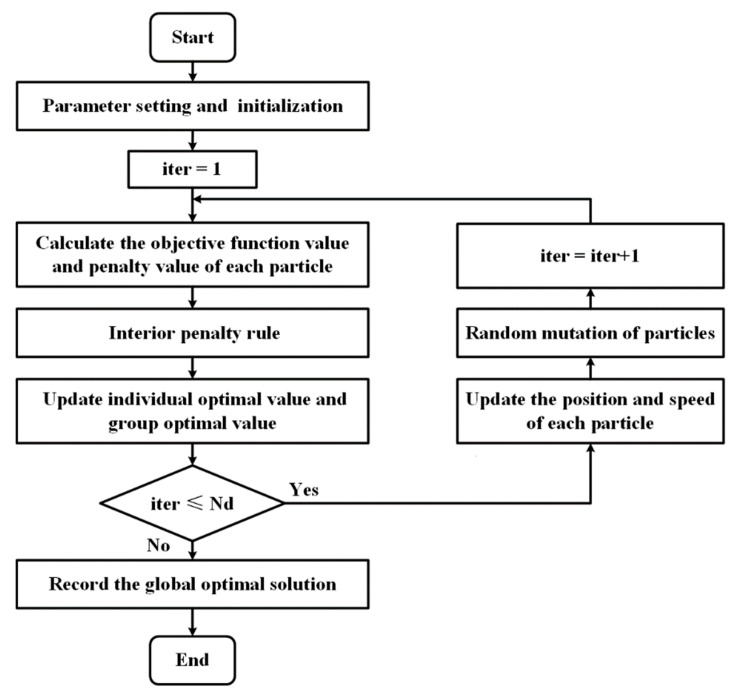
Flow chart of improved particle swarm optimization.

The complete flow of the improved particle swarm algorithm is shown in Figure 6, and the details are as follows:

Step 1: Initialize the position and velocity of each particle in the search space randomly;

Step 2: Calculate the objective function for each particle’s current position and compare it to its personal best solution;

Step 3: Update the personal and global best according to defined internal penalty function rules;

Step 4: Update the velocity and position of each particle;

Step 5: Perform random mutation operations for particles;

Step 6: Repeat steps 2–5 until a maximum number of iterations $N_{d}$ (in this paper $N_{d}=800$) is met;

Step 7: Record the global best solution as the optimization result.

### 3.3. PSO Formula for Minimum Fuel Reconfiguration Control

In order to apply the improved particle swarm optimization algorithm to solve the nonlinear programming problem described in Section 3.2, the setting of the algorithm formulation is necessary.

The PSO particle can be defined as

(26) $$X_{pso}={\left[t_{i,}{}_{1},\ldots t_{i,}{}_{2k_{i}},F_{r,}{}_{1},F_{t,}{}_{1},\ldots ,F_{r,}{}_{k_{i}},F_{t,}{}_{k_{i}},t_{o,}{}_{1}\ldots t_{o,}{}_{2k_{o}},F_{n,}{}_{1}\ldots ,F_{n,}{}_{k_{o}}\right]}^{T}$$

where $k_{i}$ and $k_{o}$ are the numbers of in-plane and out-of-plane maneuvers, respectively; $t_{i,k}(k=1,2,\ldots ,k_{i})$ are the moments of in-plane maneuvering, $\left[F_{r,}{}_{k},F_{t,}{}_{k}](k=1,2,\ldots ,k_{i}\right)$ are the thrust amplitudes of the in-plane maneuvers; $t_{o,}{}_{k}(k=1,2,\ldots ,k_{o})$ are the moments of out-plane maneuvering, and $F_{n,}{}_{k}(k=1,2,\ldots ,k_{o})$ are the thrust amplitudes of the in-plane maneuvers.

Since each variable in the orbital dynamics equations presents a large variation in the magnitude of units and physical quantities, the variables were normalized in order to avoid negative effects on the convergence of the problem, expressed as

(27) $$x_{pso}={\left[\tau_{i,}{}_{1},\ldots \tau_{i,}{}_{2k_{i}},\xi_{r,}{}_{1},\xi_{t,}{}_{1},\ldots ,\xi_{r,}{}_{k_{i}},\xi_{t,}{}_{k_{i}},\tau_{o,}{}_{1}\ldots \tau_{o,}{}_{2k_{o}},\xi_{n,}{}_{1}\ldots ,\xi_{n,}{}_{k_{o}}\right]}^{T}$$

The relationship between the normalized variables and the actual physical variables can be expressed as

$$t_{i,2k-1}=t_{i,k-1}+dt_{i,k}\tau_{i,2k-1},\;t_{i,2k}=t_{i,2k-1}+(dt_{i,}{}_{k}-t_{i,2k-1})\tau_{i,2k}\;(k=1,2,\ldots ,k_{i})$$

$$u_{r,}{}_{k}=-\frac{F_{r,k}}{m}+\frac{2F_{r,k}}{m}\xi_{r,}{}_{k},\;u_{t,}{}_{k}=-\frac{F_{t}{}_{,k}}{m}+\frac{2F_{t,k}}{m}\xi_{t,}{}_{k}\;(k=1,2,\ldots ,k_{i})$$

$$t_{o,2k-1}=t_{o,k-1}+dt_{o,k}\tau_{o,2k-1},\;t_{o,2k}=t_{o,2k-1}+(dt_{o,}{}_{k}-t_{o,2k-1})\tau_{o,2k}\;(k=1,2,\ldots ,k_{o})$$

$$u_{n,}{}_{k}=-\frac{F_{n}{}_{,k}}{m}+\frac{2F_{n}{}_{,k}}{m}\xi_{n,}{}_{k}\;(k=1,2,\ldots ,k_{o})$$

In the formula, τ,ξ∈[0,1], and m is the mass of the spacecraft. It needs to be noted that the linear inequality constraints on the maneuver times are transformed into only the bound constraints on the normalized optimization variables. In addition, for consistency with Section 3.1, the thrust amplitude is expressed as acceleration form in the new variables.

The dynamics constraint is added to the objective function as a penalty term, denoted as:

(28) $$J_{pso}=J+h_{pso}(i_{c})P_{pso}(x)$$

(29) $$P_{pso}(x)=\sum_{n=1}^{6}V(\Delta \delta \alpha_{t_{f}}(x))\Delta \delta \alpha_{t_{f}}(x)$$

(30) $$\Delta \delta \alpha_{t_{f}}(x)=\max(0,\left|\delta \alpha_{t_{f}}(x)-\delta \alpha_{des}\right|-\Delta d)$$

where $\delta \alpha_{t_{f}}(x)$ is the relative state at the end of the task, $\delta \alpha_{des}$ is the desired state, and Δd is the constraint tolerance.

The other basic settings are not described in detail here.

## 4. Numerical Simulations

This section presents the maneuver planning obtained using the improved particle swarm optimization algorithm, demonstrating the maneuvering performance in terms of delta-v, accuracy, etc.

### 4.1. Initial Values and Settings

In a selected space gravitational wave mission, the initial values of reference orbits and the errors for the three spacecraft are shown in Table 1 and Table 2, and the parameters of the satellites are shown in Table 3. The detector operates in a “3 + 3” mode, meaning that the detector conducts scientific observations for the first three months and conducts the formation reconfiguration and equipment maintenance in the following three months. During the scientific exploration, the configuration performance index of the formation should meet: the variation in arm length is less than 1732.1 km, the variation in breathing angle is less than 0.2°, and the variation in relative velocity is less than 10 m/s.

**Figure 7:**
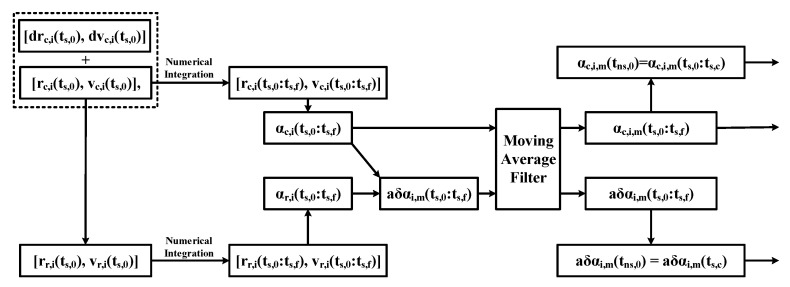
Computational sequence of the mean states.

The control method proposed in this paper is based on the mean states, whereas the measurements available to us are all osculating. Therefore, it is necessary to process the results of the numerical orbit propagation. The process is shown in Figure 7 and is briefly described as follows [37].

In the first step, the initial osculating position velocity of the real satellite is obtained from the initial reference orbit and the error is denoted as $\left[r_{r,i}(t_{s,0}),v_{r,i}(t_{s,0})\right]$. In the second step, a high-precision numerical propagation of the reference and true orbits is carried out to obtain orbital data for three months. In the third step, the orbital data obtained in the previous step is converted into osculating orbital elements. In the fourth step, the osculating relative orbital elements are calculated by Equation (3). Finally, the mean relative orbital elements and the mean orbital elements of the reference orbit through the moving average filter defined in Section 2.4 are denoted as $\delta \alpha_{c,i,m}(t_{s,0}:t_{s,f})$ and $\alpha_{c,i,m}(t_{s,0}:t_{s,f})$.

**Figure 8:**
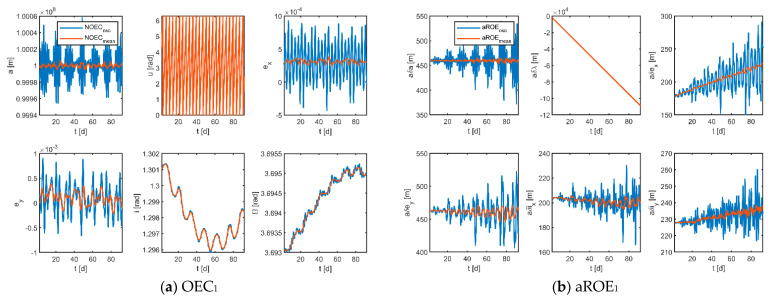

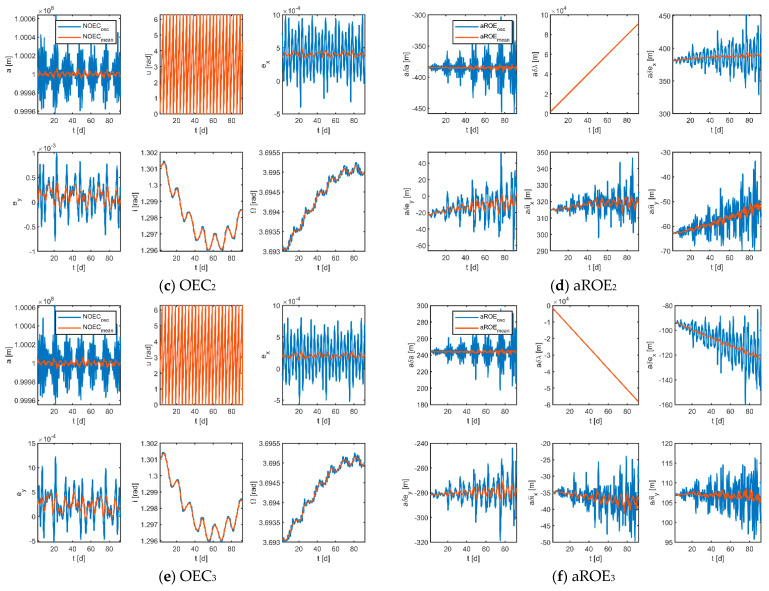
Osculating and mean states in the stage of scientific exploration (the subscripts 1, 2, 3 indicate the corresponding virtual formations).

The results of the mean states obtained by the moving average filter are shown in Figure 8. As demonstrated, the blue curves denote the osculating states and the red curves represent the mean states, which shows visually that the use of the filtering algorithm works well in removing short-term effects for both the absolute and relative orbital parameters. Furthermore, it can be deduced that if osculating ROE is used for control, excess control effort will be wasted to counter temporary effects, wasting fuel and reducing mission lifetime.

**Figure 9:**
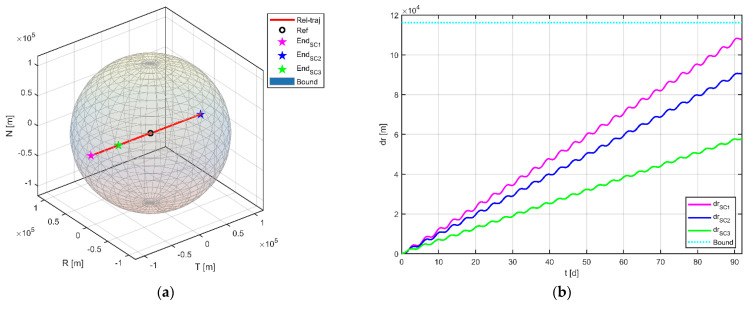
Configuration evolution of virtual formations in the process of scientific exploration. (**a**) Relative position of the satellite in the RTN coordinate system of the virtual reference point (for the brevity of the description, three virtual formations are shown in one graph); (**b**) dispersion between the physical satellites and the virtual reference point.

Furthermore, by projecting the motion of the physical spacecraft onto the RTN coordinate system of the reference point, the divergence of each virtual formation configuration can be obtained, as shown in Figure 9. In subfigure (a), it can be seen that the dispersion of the virtual formation is mainly in the tangential direction and no satellite exceeds the maximum permissible boundary of 116.16 km (calculated by Equation (2)) during the whole mission period. The divergence of each physical spacecraft with respect to the virtual reference point can be seen in subfigure (b), with maximum offsets of 108.02 km, 90.65 km, and 57.98 km for the three satellites, respectively. Judging from the dispersion, satellites 1 and 2 are close to the maximum allowable boundary of the virtual formation, so the formation reconfiguration control needs to be implemented as soon as possible after the end of the scientific observation mission.

Finally, the average relative orbital elements and the average absolute orbital elements of the reference orbit at the end of the scientific exploration phase are recorded as shown in Table 4 and Table 5, which are used as initial values for the formation reconfiguration control.

### 4.2. Validation of Linear Relative Dynamics Models

The relative dynamics model used in this paper has not yet been applied to a 100,000 km geocentric orbit, so its applicability and performance need to be discussed. It is accomplished by comparing the output of the linear model with the numerical results.

**Figure 10:**
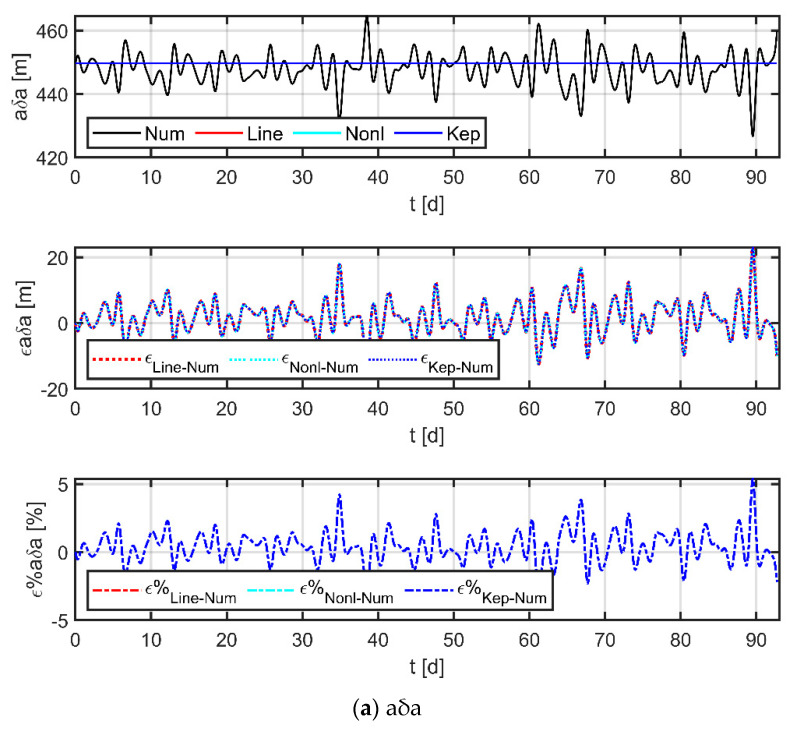

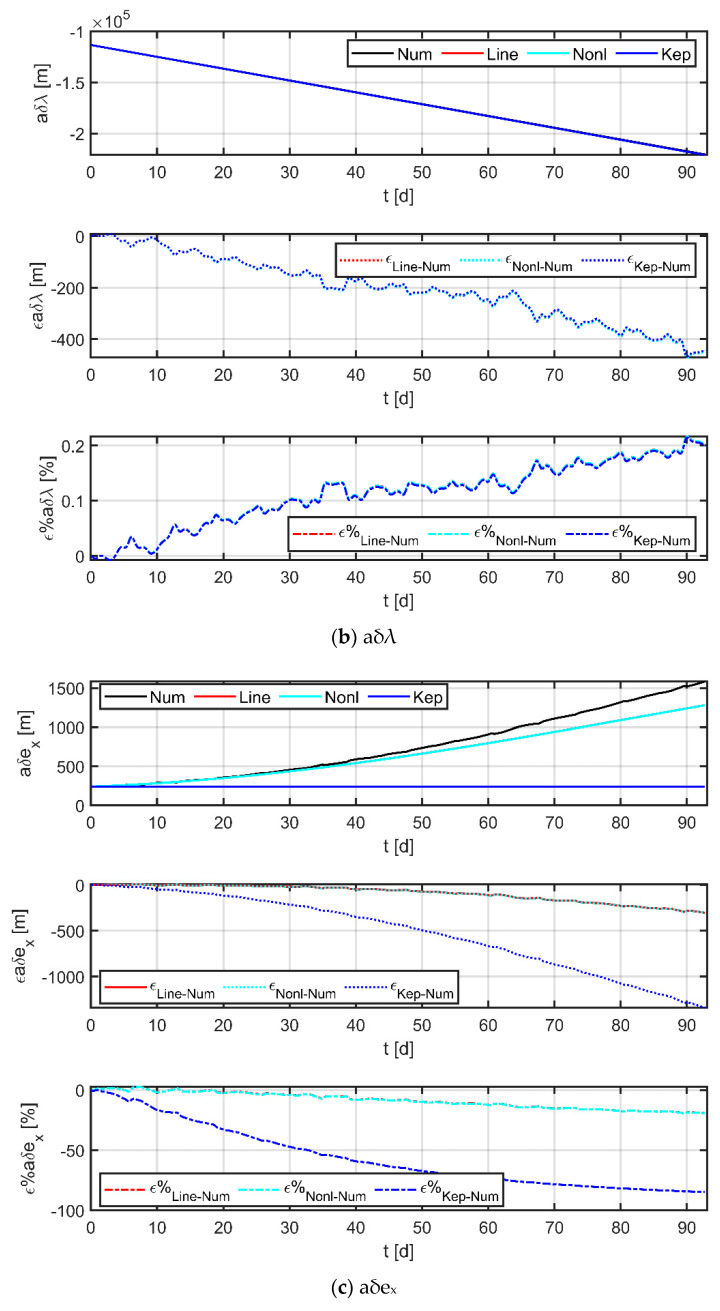

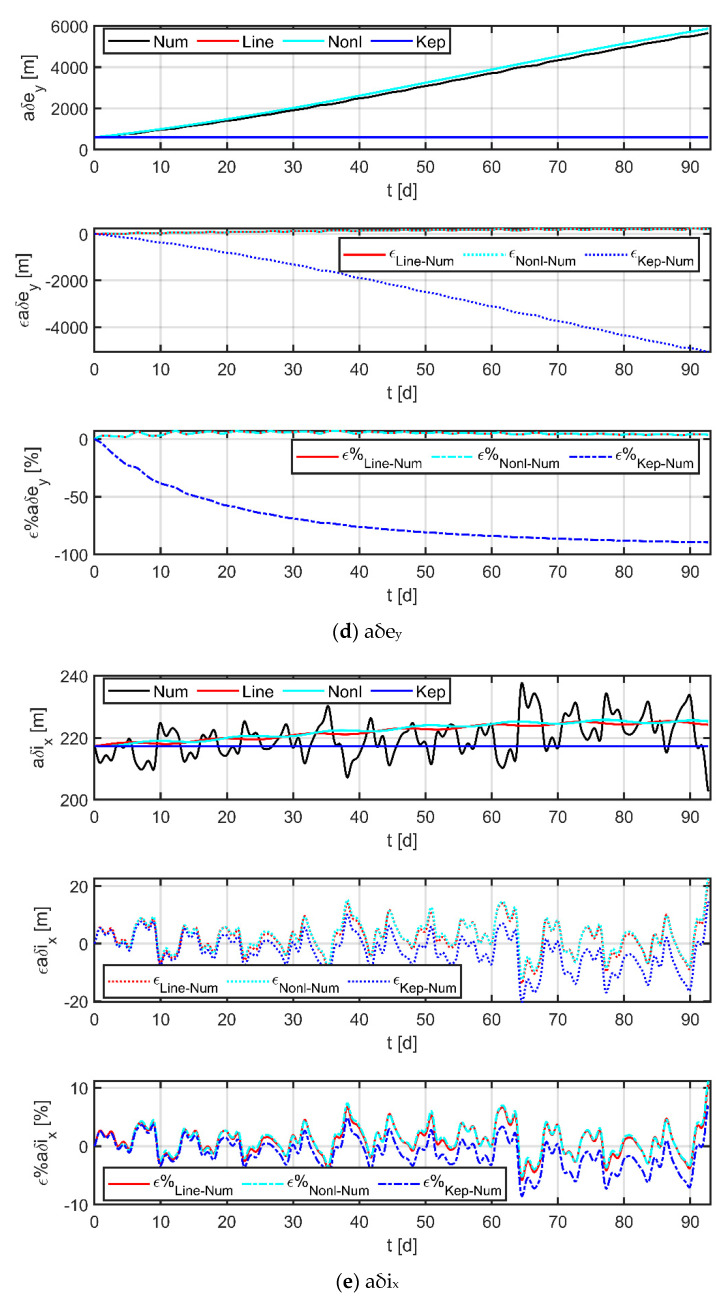

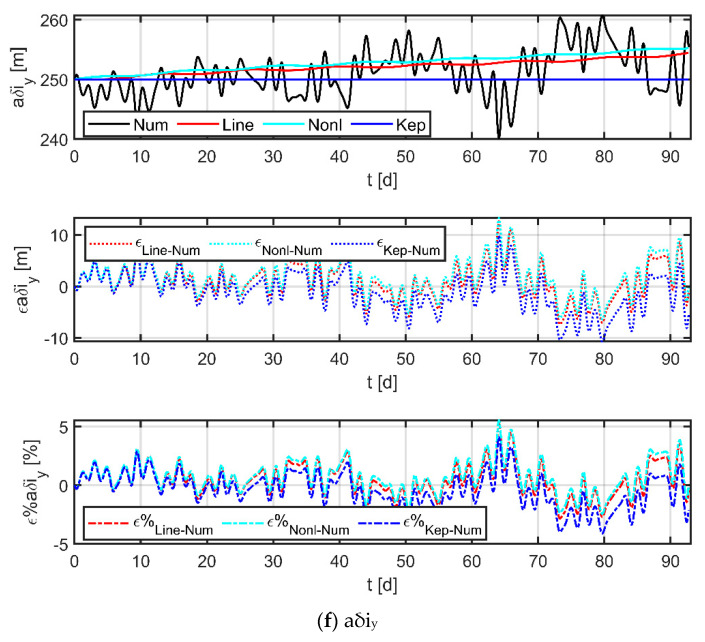
Comparison of Keplerian models, nonlinear models, linear models, and numerical results.

Assuming no drag-free control during the maintenance phase, in this case, the physical satellite is mainly affected by J_2_, SRP, and T_3rdb_ perturbations, while the virtual reference point is only affected by conservative forces such as J_2_ and T_3rdb_ since it has no physical entity. The Sc_1_ data in Table 4 and Table 5 were selected as initial values for the simulation, and simulation results are shown in Figure 10.

In the figure, the black line indicates the results obtained by numerical methods, the red line is the result of the linear model used in this paper, the cyan line is the result of the nonlinear model [38], and the blue line is the result of the Keplerian model; the three subplots from top to bottom indicate the curves of the relative orbital elements, the absolute error curve of each model with respect to the numerical results, and the relative error curve of each model with respect to the numerical results, respectively. The comparison with the Keplerian model shows that the two-body assumption does not accurately describe the orbital dynamics at 10^5^ km (mainly reflected in $e_{x}$ and $e_{y}$) and the perturbation effects are not negligible. The comparison with the nonlinear model shows that the two have almost comparable accuracy (the maximum error is less than 3 m in 92 days), which indicates that the simplification operations carried out by the linear model are valid. Furthermore, a comparison with the numerical results provides an assessment of the accuracy of the model. The statistics in Table 6 show that the maximum absolute error of the linear model is only in the order of a hundred meters over a 30-day period, and the relative errors are less than 7%; even at 60- and 90-day periods, the model still has a high level of accuracy. The largest error term occurs at $\delta e_{x}$, which is primarily due to the unmodeled terms (e.g., eclipses effects, planetary gravity, or higher-order geopotential terms), and the error can be reduced by updating the orbital elements of the reference spacecraft and the physical spacecraft within a certain time interval.

In summary, the linear model is an advantageous choice for the study of 100,000 km formation dynamics.

### 4.3. Performance Evaluation of Improved Particle Swarm Optimization Algorithms

To evaluate the performance of the improved particle swarm optimization algorithm in the paper, 20 repeated experiments were carried out with the same initial values and settings.

Assuming that the amplitudes of the three axes of the thruster were [400, 400, 200] μN, six in-plane maneuvers and four out-of-plane maneuvers were performed during the whole control process, and the given mission time was 14 days, the desired relative orbital elements are [0,0,0,0,0,0]^T^ m. The parameter settings of the algorithm include: the population scale was 1000, maximum iterations were 800; the inertia factor *W* decreased from 0.9 to 0.4, the self-learning factor $C_{1}$ decreased from 1.5 to 0.5, the group learning factor $C_{2}$ increased from 0.5 to 1.5, random mutation probability factor $\phi_{c}$ was set to 0.85, and ς was set to 35; the tolerances for dynamics constraints were [1,1,1,1,1,1]^T^ m, and the penalty factor was set as in Equation (31). The traditional particle swarm algorithm uses the same basic setup.

(31) $$V(S_{n}(x))=\{\begin{array}{cc} 0 & S_{n}(x)<0.01\\ 2 & 0.01\leq S_{n}(x)<0.05\\ 5 & 0.05\leq S_{n}(x)<0.2\\ 10 & 0.2\leq S_{n}(x)<0.5\\ 100 & 0.5\leq S_{n}(x)<1 \end{array}$$

**Figure 11:**
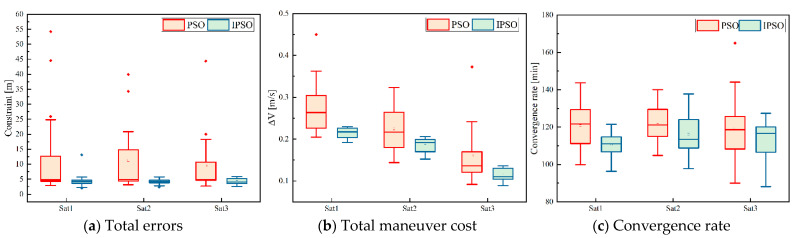
Algorithm performance comparison.

In the experimental test of the algorithm, the terminal error, maneuver cost, and convergence rate of each experiment are recorded and shown in the form of a box plot, as shown in Figure 11. A box plot is used to observe the overall distribution of the data, which is a type of chart that displays the summary of a set of statistical values with a five number summary “minimum”, first quartile (Q1), median (Q2), third quartile (Q3), and “maximum”. The “box” part of the box plot represents the interquartile range (IQR), which is the range of the middle 50% of the data, and the “whiskers” represent the minimum and maximum values, excluding outliers. The median is shown as a line inside the box and some points outside the box are anomalies. Subplot (a) shows the total errors between the terminal and desired values of the six parameters, subplot (b) shows the total cost of maneuvers during the control process, and subplot (c) shows the time consumption of the whole convergence process; the above three indicators are commonly used to describe algorithm performance.

As illustrated in subplots (a) and (b), the box representing the traditional PSO is rather long and has a high number of outliers, which indicates that traditional PSO algorithms have poor convergence and high volatility. In contrast, all boxes corresponding to the improved particle swarm algorithm are narrow and have smaller values, proving that the improved algorithm performs better and the solutions obtained are more credible. It should be noted that the advantage of subgraph (c) is not as obvious as that of subgraphs (a) and (b), mainly because the convergence rate is mainly dominated by the properties of the objective function when the solved problem is too complex.

Overall, our proposed improved PSO algorithm outperforms the traditional PSO in terms of both convergence precision and convergence rate.

### 4.4. Fuel-Minimum Reconfiguration Maneuver Planning via IPSO

Assuming that the amplitude of the three axes of the thruster is [400, 400, 200] μN, six in-plane maneuvers, and four out-of-plane maneuvers are performed during the whole reconfiguration process, the given mission time is 14 days, the desired relative orbital elements are [0,0,0,0,0,0]^T^ m, and the tolerances of the six relative orbital elements are [1,1,1,1,1,1]^T^ m. In addition, set [3.1, 3.5] d, [11.5, 11.9] d as the maintenance phase of the instruments, during which the satellites are not available for formation control.

**Figure 12:**
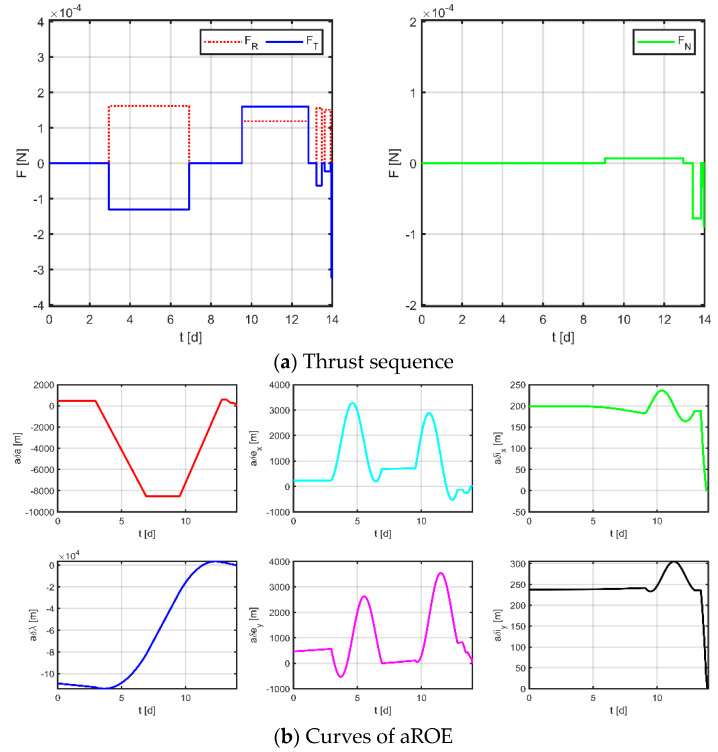

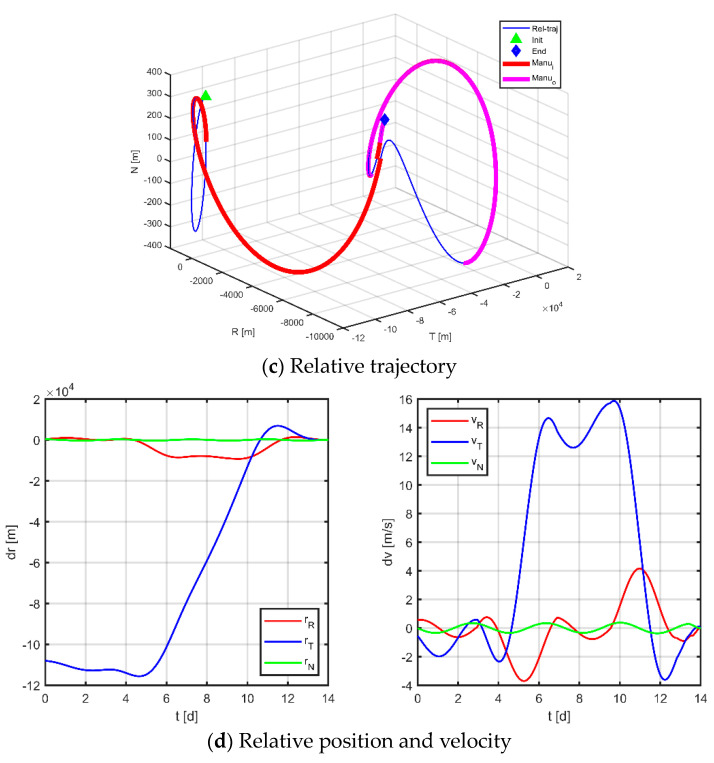
Maneuvering solution for virtual formation 1 via general strategy.

**Figure 13:**
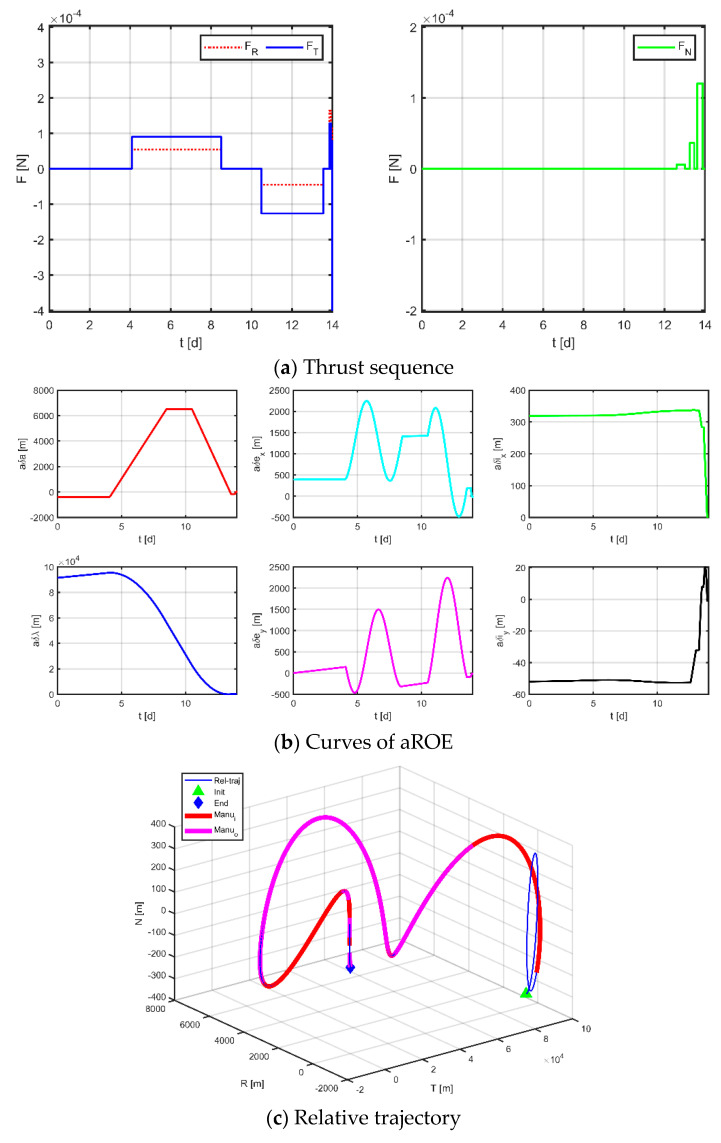

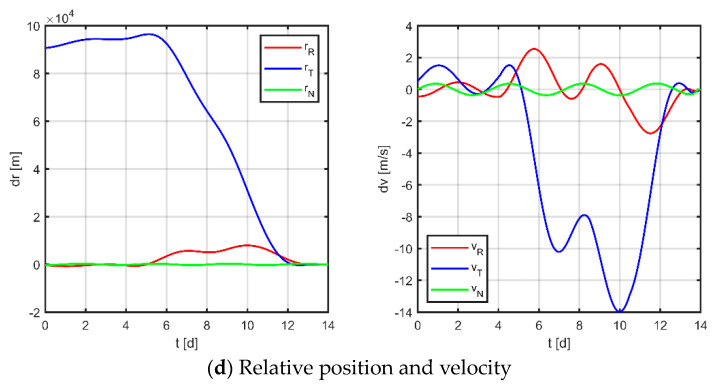
Maneuvering solution for formation 2 via general strategy.

**Figure 14:**
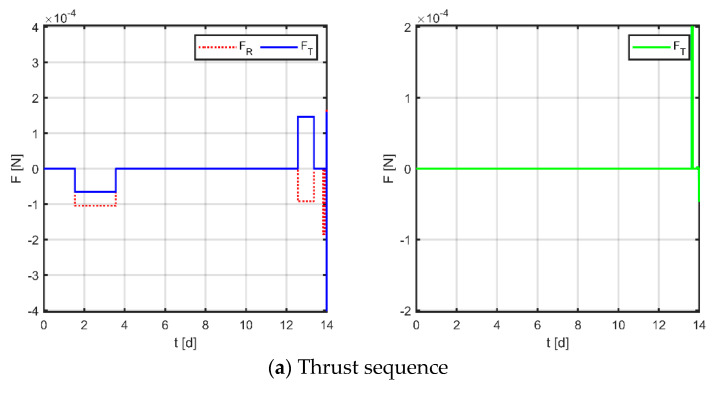

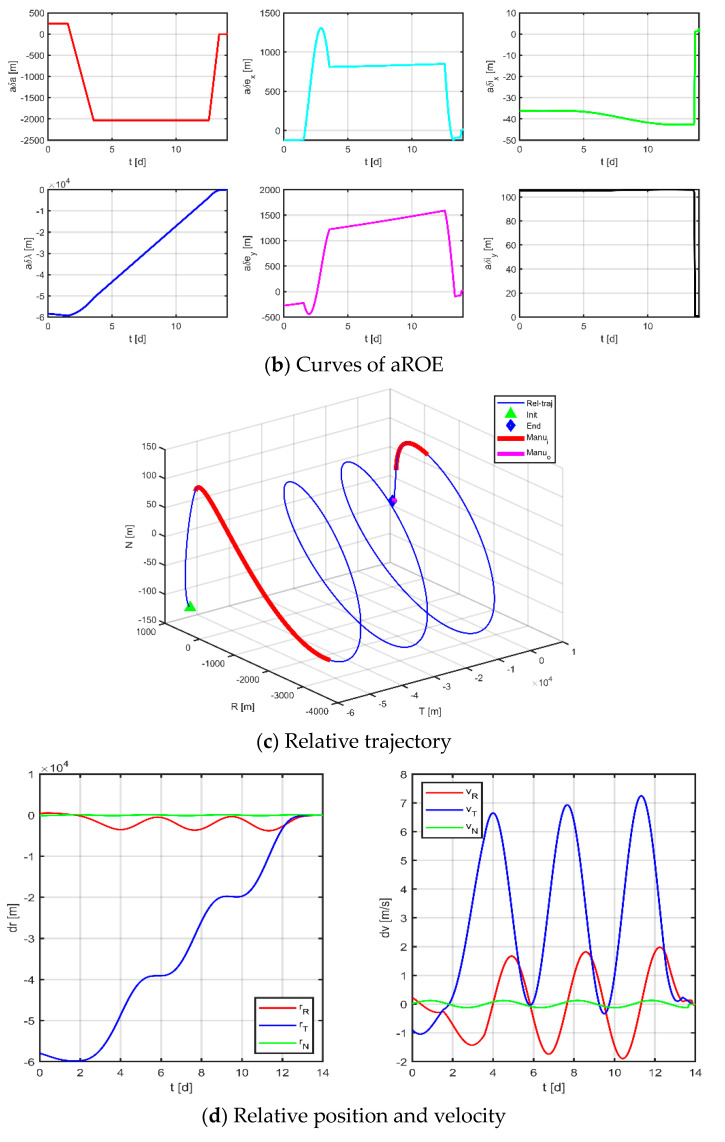
Maneuvering solution for formation 3 via general strategy.

Firstly, as a comparison term to the strategy proposed in this paper, the near-Earth formation reconstruction strategy of the literature [23] is directly transposed to the space gravitational wave mission, without discretization of the mission space and without normalization of the optimization variables. Simulation results are shown in Figure 12, Figure 13 and Figure 14, where subplot (a) is the optimized maneuver sequence, subplot (b) is the curves of the relative orbital elements throughout the control process, subplot (c) is the relative trajectory in the RTN coordinate system of the virtual reference spacecraft, and subplot (d) is the relative position and relative velocity curve in the virtual reference spacecraft RTN coordinate system. Subplots (c) and (d) are transformed by substituting the relative orbit element data into Equation (5), whose end states reflect the accuracy of the spacecraft tracking the reference trajectory.

The results for virtual formation 1 are shown in Figure 12. For in-plane motion, the maneuvers’ times are [2.951, 6.914, 9.532, 12.824, 13.224, 13.478, 13.628, 13.912, 13.951, 13.978, 13.989, 13.997] d, the maneuvers’ magnitudes along the R-axis are [161.368, 118.499, 155.670, 150.948, 4.266, −48.326] μN, and the maneuvers’ magnitudes along the T-axis are [−131.078, 159.712, −63.487, −22.802, −322.770, −198.324] μN. For out-of-plane motion, the maneuvers’ times are [9.073, 12.946, 13.414, 13.831, 13.831, 13.920, 13.964, 13.966] d, and the magnitudes of the maneuvers along the N-axis are [7.101, −78.100, −34.080, −89.897] μN. Correspondingly, the total maneuvers’ cost is 0.3895 m/s, and the relative orbital elements converge to [0.981, −0.508, −0.671, −0.984, −0.999, 0.996]. The terminal relative position error is [2.008, −1.725, −1.064] m, and the terminal relative velocity error is [−0.667, −3.390, 1.024] mm/s.

The results for virtual formation 2 are shown in Figure 13. For in-plane motion, the maneuvers’ times are [4.082, 8.504, 10.492, 13.548, 13.847, 13.922, 13.970, 13.987, 13.993, 13.996, 13.999, 13.999] d, the maneuvers’ magnitudes along the R-axis are [54.630, −45.172, 163.718, 114.186, 16.520, 65.087] μN, and the maneuvers’ magnitudes along the T-axis are [90.019, −126.130, 128.242, 78.819, −399.198, −36.733] μN. For out-of-plane motion, the maneuvers’ times are [5.900, 10.913, 12.594, 13.018, 13.255, 13.466, 13.616, 13.891] d, and the magnitudes of the maneuvers along the N-axis are [0, 5.697, 36.544, 120.304] μN. Correspondingly, the total maneuvers’ cost is 0.2130 m/s, and the relative orbital elements converge to [0.977, −0.830, 0.995, −0.973, 0.990, −0.980]. The terminal relative position error is [1.441, −3.454, −1.315] m, and the terminal relative velocity error is [−1.459, −2.647, 0.506] mm/s.

The results for virtual formation 3 are shown in Figure 14. For in-plane motion, the maneuvers’ times are [1.544, 3.564, 12.564, 13.365, 13.810, 13.891, 13.976, 13.982, 13.988, 13.988, 13.992, 14] d, the maneuvers’ magnitudes along the R-axis are [−104.884, −91.683, −184.478, 167.947, 141.187, 160.740] μN, and the maneuvers’ magnitudes along the T-axis are [−65.187, 146.413, −1.056, 158.838, −400.000, −67.483] μN. For out-of-plane motion, the maneuvers’ times are [13.628, 13.694, 13.880, 13.970, 13.990, 13.993, 13.994, 13.997] d, and the magnitudes of the maneuvers along the N-axis are [200.000, 1.824, −15.123, −45.862] μN. Correspondingly, the total maneuvers’ cost is 0.0979 m/s, and the relative orbital elements converge to [0.964, −0.278, 0.705, 0.472, 0.879, 0.0979]. The terminal relative position error is [−0.641, −1.847, −1.268] m, and the terminal relative velocity error is [−0.867, −0.847, 2.290] mm/s.

**Figure 15:**
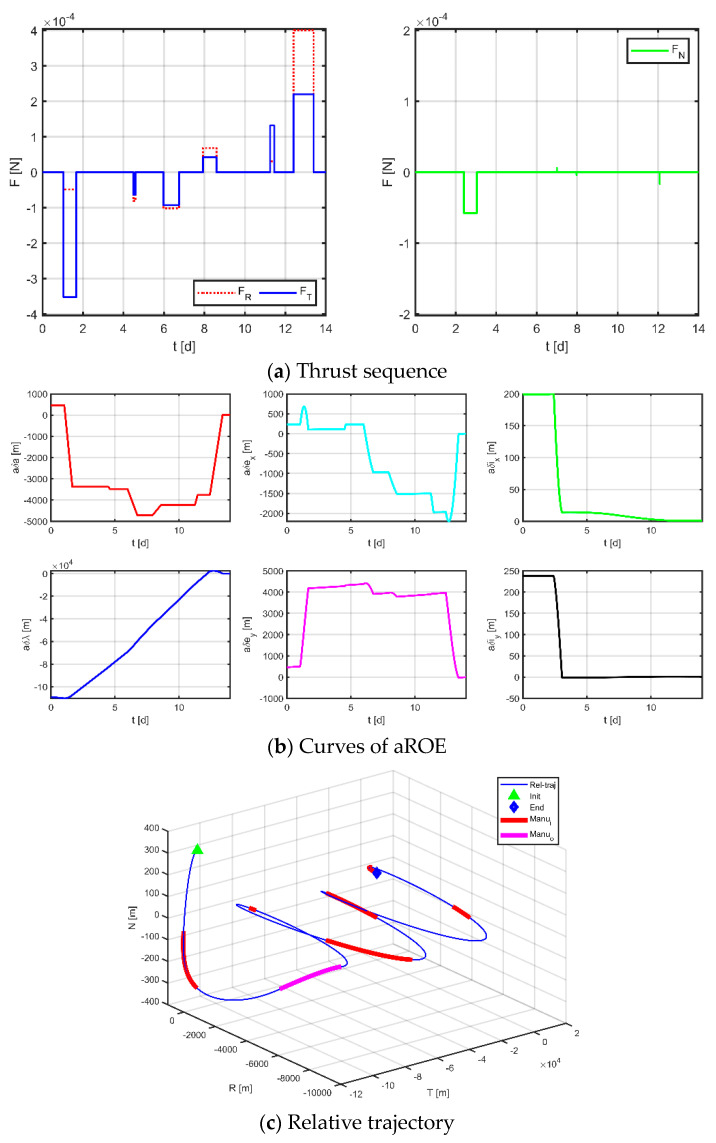

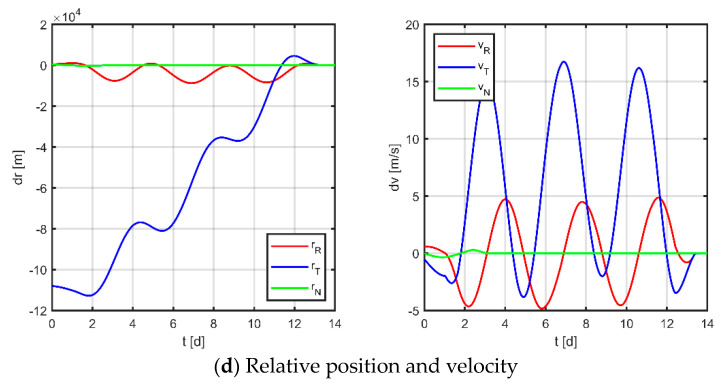
Maneuvering solution for formation 1 via discrete strategy.

**Figure 16:**
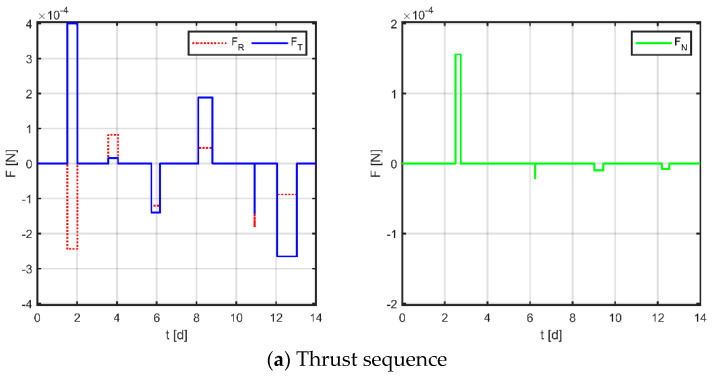

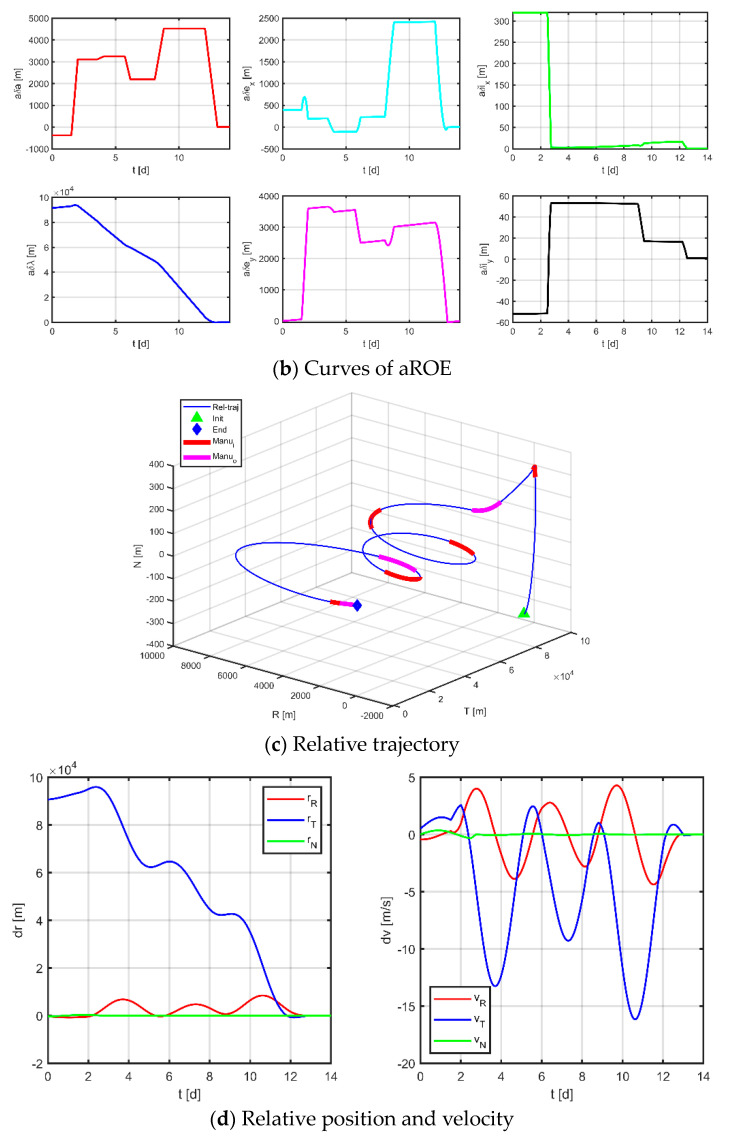
Maneuvering solution for formation 2 via discrete strategy.

**Figure 17:**
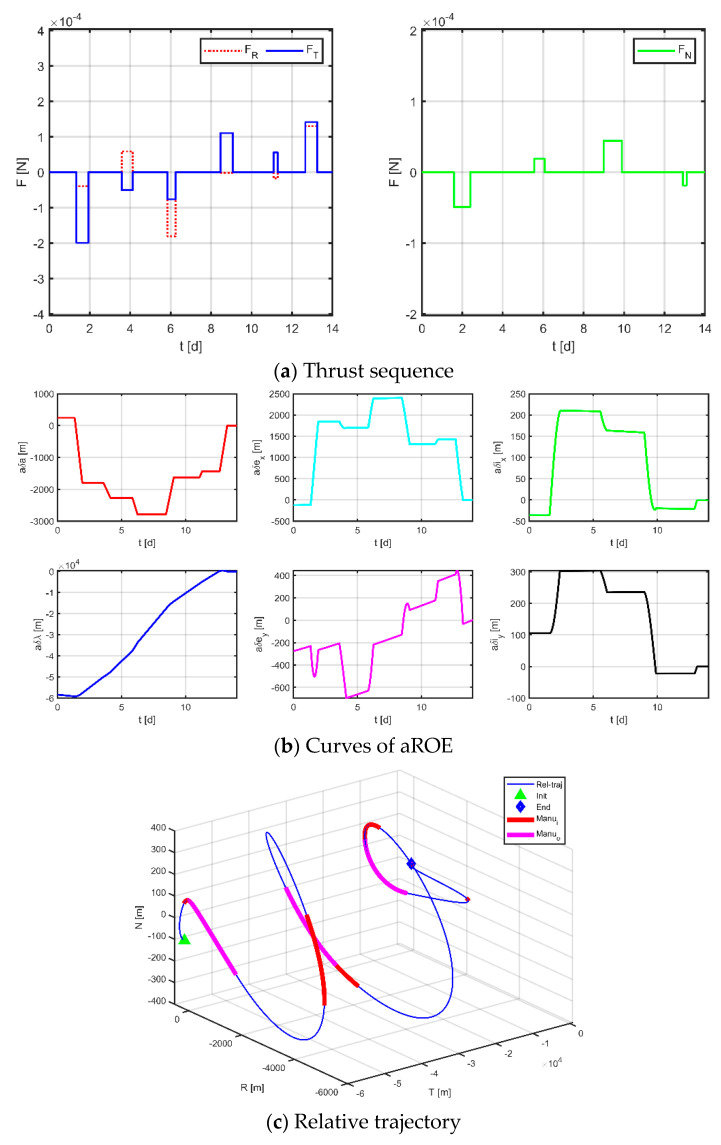

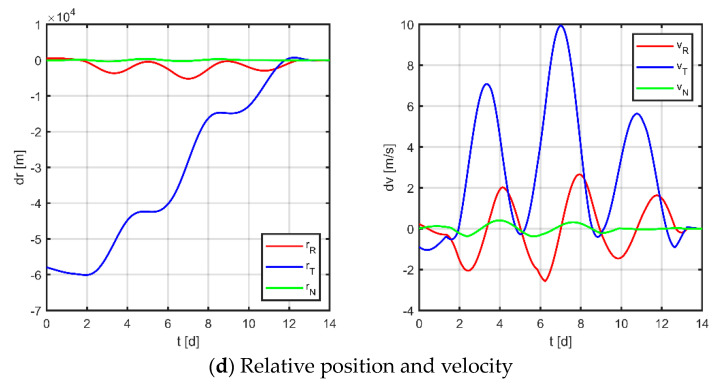
Maneuvering solution for formation 3 via discrete strategy.

Secondly, applying the method proposed in Section 3.1 to the discrete entire task interval, each subinterval of the in-plane is [0, 3.10] d, [3.50, 4.67] d, [4.67, 7.00] d, [7.00, 9.33] d, [9.33, 11.50] d, [11.90, 14] d; each subinterval of the out-of-plane is [0, 3.10] d, [3.50, 7.00] d, [7.0, 11.50] d, [11.90, 14.00] d, and only one maneuver is allowed in each interval. Figure 15, Figure 16 and Figure 17 demonstrate the simulation results.

The results for virtual formation 1 are shown in Figure 16. For in-plane motion, the maneuvers’ times are [1.037, 1.665, 4.494, 4.589, 5.977, 6.743, 7.931, 8.596, 11.246, 11.452, 12.414, 13.400] d, the maneuvers’ magnitudes along the R-axis are [−48.437, −81.167, −102.014, 68.190, 30.341, 400.000] μN, and the maneuvers’ magnitudes along the T-axis are [−352.682, −63.399, −93.112, 41.924, 131.971, 220.195] μN. For out-of-plane motion, the maneuvers’ times are [2.409, 3.047, 7.000, 7.000, 7.972, 7.972, 12.081, 12.081] d, and the magnitudes of the maneuvers along the N-axis are [−57.567, 6.132, −4.178, −16.619] μN. Correspondingly, the total maneuvers’ cost is 0.2023 m/s, and the relative orbital elements converge to [−0.383, −0.920, −0.954, −0.909, 0.999, 0.998] m. The terminal relative position error is [0.588, −2.702, 0.931] m, and the terminal relative velocity error is [−0.985, −1.512, 1.176] mm/s.

The results for virtual formation 2 are shown in Figure 17. For in-plane motion, the maneuvers’ times are [1.507, 2.011, 3.568, 4.056, 5.732, 6.167, 8.091, 8.804, 10.925, 10.925, 12.056, 13.040] d, the maneuvers’ magnitudes along the R-axis are [−104.884, −91.683, −184.478, 167.947, 141.187, 160.740] μN, and the maneuvers’ magnitudes along the T-axis are [400.000, 15.861, −140.128, 188.800, −141.672, −265.133] μN. For out-of-plane motion, the maneuvers’ times are [2.499, 2.747, 6.241, 6.242, 9.016, 9.450, 12.196, 12.541] d, and the magnitudes of the maneuvers along the N-axis are [155.973, −211.180, −9.594, −7.557] μN. Correspondingly, the total maneuvers’ cost is 0.1806 m/s, and the relative orbital elements converge to [−0.997, 0.328, 0.999, −0.733, 0.970, 0.935]. The terminal relative position error is [−0.423, −1.868, 0.411] m, and the terminal relative velocity error is [−1.214, 0.385, −1.418] mm/s.

The results for virtual formation 3 are shown in Figure 17. For in-plane motion, the maneuvers’ times are [1.229, 1.929, 3.581, 4.125, 5.847, 6.241, 8.469, 6.241, 8.469, 9.077, 11.091, 11.290, 12.661, 13.246] d, the maneuvers’ magnitudes along the R-axis are [−39.302, 58.738, 180.544, −1.430, −17.756, 130.166] μN, and the maneuvers’ magnitudes along the T-axis are [−199.369, −50.448, −76.154, 110.663, 55.742, 141.606] μN. For out-of-plane motion, the maneuvers’ times are [1.585, 2.390, 5.554, 6.074, 8.988, 9.892, 12.915, 13.096] d, and the magnitudes of the maneuvers along the N-axis are [−49.114, 18.886, 44.440, −18.813] μN. Correspondingly, the total maneuvers’ cost is 0.1100 m/s, and the relative orbital elements converge to [0.768, −0.622, 0.349, 1.000, −0.995, −0.435]. The terminal relative position error is [1.036, −2.671, 0.916] m, and the terminal relative velocity error is [−1.133, −1.865, −0.646] mm/s.

Comparing the results of the two approaches, it can be seen that both methods can obtain maneuver solutions that satisfy the dynamical constraints; however, there are some differences in the maneuver cost and maneuver distribution. Firstly, in terms of the maneuver cost, theoretically analyzed, the conventional method does not constrain the complete solution space and is supposed to obtain a relatively better solution, but from the actual results, the delta-v consumptions of the three satellites obtained by the two methods are [0.3895, 0.2130, 0.0979] m/s and [0.2030, 0.1806, 0.1100] m/s, respectively. The proposed method performs better instead. This phenomenon may be caused by two reasons: one because the algorithm easily falls into local optimum due to a large number of variables and complex constraints of the problem under study; another is that the conventional method does not normalize the variables, which is not beneficial to the convergence of the solution. Second, in terms of the distribution of maneuver profiles, the maneuver profiles obtained using the conventional method tend to impose two maneuvers of longer duration followed by a series of short-duration maneuvers for quick adjustment in the final stage. In contrast, the maneuver solutions obtained by the method proposed in this paper are more uniform regarding the location and duration of each maneuver due to the discretization of the mission time. Finally, in terms of accuracy, both methods have comparable accuracy, and the maximum relative position error is no more than 4 m and the maximum relative velocity error is no more than 4 mm/s. It should be noted that some factors in the paper (e.g., random variation probability, inertia factor, learning factor, penalty factor, etc.) are set empirically, which will affect the performance of the algorithm to some extent; in addition, the discretization method of the whole task interval will also affect the acquisition of the optimal solution.

To verify the universality of the proposed method, Table 7 and Table 8 are obtained by applying the method to different thruster schemes and different given mission times. In different thruster schemes, the mission time is limited to 14 d; in different given mission time schemes, the thruster amplitude is given as [400, 400, 200] μN. The simulation results show that the proposed method is well-suited and flexible enough to meet the possible formation reconfiguration requirements in space gravitational wave missions. In addition, the data in Table 7 show that the total delta-v does not present a clear regularity as the thruster amplitude changes. The data in Table 8 show that the total delta-v generally tends to decrease and then increases with the gradual increase in the given mission time, which is because part of the offset of δλ can be corrected by the natural dynamics established by δa, the divergence dominating again with the prolongation of the task time. It should be noted that the above analysis is limited to the phenomenon presented in this study and cannot be taken as a general rule.

## 5. Conclusions

This paper addressed the design of the fuel-minimum maneuvering strategy for the formation reconfiguration in the high earth orbit (10^5^ km). In this study, a control strategy utilizing virtual formation is employed to address the issue of measurement-limited formation. The relative motion of real satellites relative to a virtual reference point is described by a linear dynamic model, which encompasses J_2_, SRP, and the third-body gravitational effects of the Sun and Moon. A piecewise continuous control strategy is chosen and the entire mission interval is discretized in the reconfiguration process to avoid persistent or high-frequency orbital maneuver disturbances to the satellite platform. The minimum fuel reconfiguration problem is transformed into a constrained nonlinear optimization problem, and an improved particle swarm algorithm is proposed for maneuver planning solutions.

Simulation results indicate that the improved particle swarm optimization algorithm has better stability and superior global search capability, and the proposed reconfiguration control strategy can flexibly respond to different complex scenarios during gravitational wave missions with less interference to the satellite platform. In addition, the maximum relative position error is only 3.454 m and the maximum relative velocity error is only 3.390 mm/s, which can satisfy the precision requirements of space gravitational wave formation maintenance and reconfiguration.

Future work will include developing more efficient optimization algorithms, and improving the dynamical models used to derive multi-objective optimization strategies, as well as extending the continuous scheme to orbits of arbitrary eccentricity and to various space missions.

## Figures and Tables

**Table 1:** Initial values of the reference orbits [36] (In the Earth J2000 ECI frame) at the epoch 22 May, 2034 12:00:00 UTC.

	x (km)	y (km)	z (km)	v_x_ (km/s)	v_y_ (km/s)	v_z_ (km/s)
S_c1_	−46,746.087307	−51,973.844583	71,473.835818	1.448401	0.471646	1.291321
S_c2_	86,220.582041	46,448.360669	20,269.217366	0.085035	0.663048	−1.881140
S_c3_	−39,378.654985	5547.379475	−91,728.424823	−1.533416	−1.134792	0.590239

**Table 2:** Initial errors of each satellite. (In the Earth J2000 ECI frame).

	dx (m)	dy (m)	dz (m)	dv_x_ (m/s)	dv_y_ (m/s)	dv_z_ (m/s)
S_c1_	5	1	1	0.002	0.008	0.002
S_c2_	−8	2	−4	0.004	−0.006	0.002
S_c3_	−6	−3	−2	−0.002	−0.003	−0.003

**Table 3:** Parameters of the satellite.

Mass (kg)	Cross-Section Area (m^2^)	Reflectance Coefficient
500	1	1.15

**Table 4:** Initial mean relative orbit elements in the formation control stage (In the Earth J2000 ECI frame) at the epoch 22 August, 2034 12:00:00 UTC.

	aδa (m)	aδλ (m)	aδe_x_ (m)	aδe_y_ (m)	aδi_x_ (m)	aδi_y_ (m)
S_c1_	463.040013	−109.045018 × 10^3^	229.276224	463.022508	198.974764	237.667251
S_c2_	−382.492916	91.458799 × 10^3^	391.707168	1.872138	319.425055	−51.944995
S_c3_	243.597393	−58.381609 × 10^3^	−122.665065	−276.252985	−36.235340	105.330778

**Table 5:** Initial mean orbit elements of virtual reference points in the formation control stage (In the Earth J2000 ECI frame) at the epoch 22 August, 2034 12:00:00 UTC.

	a (km)	u (rad)	e_x_	e_y_	i (rad)	Ω (rad)
S_c1_	100,002.493442	2.487123	2.952623 × 10^−4^	−8.979140 × 10^−4^	1.298356	3.694982
S_c2_	99,998.851263	4.581601	3.767485 × 10^−4^	4.5754951 × 10^−5^	1.298381	3.694975
S_c3_	99,999.388761	0.392801	2.1307589 × 10^−4^	2.040820 × 10^−4^	1.298378	3.694990

**Table 6:** Error statistics for the linear model.

	aδa		aδλ		aδe_x_		aδe_y_		aδi_x_		aδi_y_	
Time (d)	ϵ_max_ (m)	σϵ_max_ (%)	ϵ_max_ (m)	σϵ_max_ (%)	ϵ_max_ (m)	σϵ_max_ (%)	ϵ_max_ (m)	σϵ_max_ (%)	ϵ_max_ (m)	σϵ_max_ (%)	ϵ_max_ (m)	σϵ_max_(%)
10	9.220	2.093	41.515	0.035	7.166	2.565	50.262	6.325	8.726	4.463	7.552	3.134
30	10.061	2.289	151.846	0.102	18.280	4.253	116.797	6.990	8.726	4.463	7.552	3.134
60	18.185	4.215	253.463	0.139	111.762	12.418	184.143	7.018	13.955	7.354	7.552	3.134
90	22.935	5.375	470.805	0.217	293.552	19.318	228.539	7.018	14.045	7.354	12.624	5.539

**Table 7:** Maneuver consumption for different thruster schemes.

Thrust Magnitude (μN)	Formation 1		Formation 2		Formation 3	
	ϵ_tol_ (m)	ΔV (m/s)	ϵ_tol_ (m)	ΔV (m/s)	ϵ_tol_ (m)	ΔV (m/s)
[100,100,100]	5.3416	0.1913	4.9592	0.1449	4.3922	0.1125
[200,200,100]	4.5372	0.1828	4.3195	0.1570	5.4405	0.0976
[200,200,200]	4.4133	0.2115	4.8920	0.1740	3.2860	0.1069
[400,400,100]	4.7147	0.2182	5.2438	0.1457	3.6645	0.1258
[400,400,200]	5.1630	0.2023	4.9620	0.1806	4.1690	0.1100
[400,400,400]	3.7862	0.2319	5.6897	0.1692	5.8692	0.0863

**Table 8:** Maneuver consumption for different task periods.

Task Period (d)	Formation 1		Formation 2		Formation 3	
	ϵ_tol_ (m)	ΔV (m/s)	ϵ_tol_ (m)	ΔV (m/s)	ϵ_tol_ (m)	ΔV (m/s)
5	4.4205	0.4604	5.9173	0.4340	5.4229	0.2214
8	4.7063	0.2963	3.9127	0.2480	3.3193	0.1388
14	5.1630	0.2023	4.9620	0.1806	4.1690	0.1100
20	4.9569	0.1786	5.7545	0.1612	4.5672	0.0876
26	5.7286	0.1620	5.3368	0.1654	5.6815	0.1035
32	3.5114	0.2261	3.9431	0.1785	5.9684	0.0923

## Data Availability

Not applicable.

## Nomenclature

$S_{ci}$ $,\;S_{ri}$	Virtual reference spacecraft and physical spacecraft
$L_{ij}$	Arm length between spacecraft $S_{ri}$$\;\mathrm{and}\;S_{rj}$
$A_{i}$	$\mathrm{Breathing}\;\mathrm{angle}\;\mathrm{corresponding}\;\mathrm{to}\;\mathrm{spacecraft}\;S_{ri}$
Δr	Envelope radius of the virtual formation
ΔL	Arm length variation index
ΔA	Breathing angle variation index
$L_{0}$ $,\;L_{\min}$ $,\;L_{\max}$	Nominal, minimum, maximum arm length, respectively
$A_{0}$ $,\;A_{\min}$ $,\;A_{\max}$	Nominal, minimum, maximum breathing angle, respectively
α	Classical Keplerian orbital elements
δα	Quasi-nonsingular relative orbital elements
u	Mean argument of latitude
φ	Relative argument of the apogee angle
θ	Relative ascending node
δx	Relative position and velocity
$W_{c}$	Mean motion of the reference orbit
$A_{kep}$ $,\;A_{J_{2}}$ $,\;A_{srp}$ $,\;A_{3rdb}$	Plant matrix of Kepler, *J*_2_, SRP, and lunisolar third-body
B	Control input matrix
$u_{r}$ $,\;u_{t}$ $,\;u_{n}$	Control acceleration component
$B_{srp}$	Ballistic coefficient
$R_{E}$	Earth radius
$P_{sun}$	Solar flux at 1 AU from the Sun
c	Light speed
$C_{r}$	Reflectivity coefficient
$A_{srp}$	Cross-sectional area perpendicular to the Sun’s direction
m	Satellite mass
ε	Ecliptic obliquity
α	Inclination of the lunar orbit to the ecliptic
$\lambda_{s}$	Mean ecliptic longitude of the Sun
$\lambda_{m}$	Mean longitude of the Moon
J	Total maneuver cost
F(x)	Generalized objective function
h	Penalty factor
$i_{c}$	Current generation
P(x)	Penalty term of the constraint
$c_{1},c_{2},\ldots c_{i}$	Penalty values
$q_{1},q_{2},\ldots q_{i-1}$	Tolerances
W	Inertia coefficient
$C_{1}$	Self-learning factor
$C_{2}$	Group learning factor
dim	Dimension of the optimization variable
x , v	Position and velocity of the particle
$p_{\mathrm{best}\;}$	Optimal solution of the particle itself
$g_{\mathrm{best}\;}$	Global optimal solution
ς	Empirical parameter
$r_{d}$	Random number distributed between 0 and 1
$\phi_{c}$	Mutation probability
t	Maneuver time
F	Maneuver magnitude
τ and ξ	Normalized variables
$\delta \alpha_{des}$	Desired relative state
Δd	Dynamics constraint tolerance
